# Functional analysis of *NtPDX2* in *Nicotiana tabacum* L. associated with stem development

**DOI:** 10.3389/fpls.2025.1547677

**Published:** 2025-04-22

**Authors:** Li Xu, Yuxin Cui, Jiaxin Xing, Qili Mi, Zhixing Wang, Xujing Wang, Wanli Zeng, Haiying Xiang, Jiarui Jiang, Lele Deng, Kunmiao Wang, Jiangtao Yang, Qian Gao

**Affiliations:** ^1^ Technology Center of China Tobacco Yunnan Industrial Co. Ltd., Kunming, China; ^2^ Biotechnology Research Institute, Chinese Academy Agricultural Sciences, MOA Key Laboratory on Safety Assessment (Molecular) of Agri-GMO, Beijing, China

**Keywords:** *Nicotiana tabacum* L., plant height, stem thickness, vitamin B_6_, *NtPDX2*

## Abstract

Vitamin B_6_ is a water-soluble vitamin that is essential for all living organisms in their life activities. Among its forms, pyridoxal 5’-phosphate (PLP) is the primary metabolically active form of Vitamin B_6_, which usually plays a crucial role in the metabolism of proteins, fatty acids, and carbohydrates. To date, although the molecular functions of genes involved in vitamin B_6_ biosynthesis, including *Pdx1*, *Pdx2*, *Pdx3*, and *Sos4*, have been reported in various plants, no studies have yet explored the functions of *NtPDX1* and *NtPDX2* in tobacco. This study used the *Nicotiana tabacum* L. as material to clone the CDS sequence of the *NtPDXs*. Through bioinformatics analysis, we predicted the phylogenetic relationships and functions of these genes; the subcellular localization of *NtPDX2* was found to be in the cytoplasmic structures. By conducting both constitutive overexpression and homozygous knockout studies of the *NtPDX2*, we observed a significant increase in vitamin B_6_ content in the stem tissues of overexpressing plants (up to 150%), while knockout plants showed a decrease to 60%. This led to changes in agronomic traits such as plant height and stem thickness in tobacco plants. The overexpressing plants exhibited a significant increase in height (100.93 cm) and stem thickness (13.64 cm), whereas the knockout plants were shorter in height (73.10 cm) and had thinner stems (10.83 cm). By integrating transcriptome sequencing technology with molecular biology methods, we aim to elucidate the molecular mechanisms underlying the role of *NtPDX2* in tobacco growth and development, thereby providing new genetic resources and a theoretical foundation for the cultivation of new tobacco varieties with superior quality for flue-cured tobacco.

## 1 Introduction

Vitamin B_6_ is an essential water-soluble vitamin that plays a critical role in the life activities of all living organisms. Within biological systems, it is present in multiple forms, with six primary forms being pyridoxal (PL), pyridoxine (PN), pyridoxamine (PM), and their 5′-phosphorylated derivatives: pyridoxal 5′-phosphate (PLP), pyridoxine 5′-phosphate (PNP), and pyridoxamine 5′-phosphate (PMP) (Fitzpatrick et al., 2007). PLP, the phosphorylated derivative of vitamin B_6_, typically acts as a coenzyme and is involved in over 200 enzymatic reactions within cells, significantly contributing to the metabolism of proteins, fatty acids, and carbohydrates (Colinas et al., 2016). However, mammals lack the ability to synthesize vitamin B_6_ *de novo*, rendering plants one of the primary sources of this vitamin in their diet (Fitzpatrick, 2011).

In plants, pyridoxal 5′-phosphate (PLP) is primarily biosynthesized *de novo* through the “DXP-independent pathway,” initially discovered in Arabidopsis thaliana (Tambasco-Studart et al., 2005). In the *de novo* biosynthesis pathway of PLP, two glutamine transferases, PDX1 and PDX2, are required (Moccand et al., 2011). Specifically, the PDX2 subunit acts as an ammonium donor, catalyzing the generation of ammonium ions from glutamine and transferring them to PDX1, while PDX1 serves as an ammonium receptor and synthase, responsible for incorporating the provided ammonium with other substrates to synthesize PLP (Mangel et al., 2019). Additionally, it has been found that PLP can be generated through the interconversion of different forms of vitamin B_6_ via the salvage synthesis pathway, which is present in all living organisms (Colinas and Fitzpatrick, 2015). The main enzymes involved this pathway include SOS4 (salt overly sensitive 4, a PMP/PNP/PLP kinase), PLR1 (PL reductase), and PDX3 (PMP/PNP oxidase), among others (Colinas et al., 2016).

A wealth of research has shown that genetic engineering techniques can successfully enhance the vitamin B_6_ levels in plants. For instance, the simultaneous overexpression of *AtPDX1.1* and *AtPDX2* in both Arabidopsis and cassava has led to a notable elevation in vitamin B_6_ content. This increase is observed in the leaves and seeds of the genetically modified Arabidopsis, as well as in the leaves and roots of the transgenic cassava cultivated in field conditions (Raschke et al., 2011; Li et al., 2015). In potatoes, constitutive overexpression of *AtPDX2* also leads to a significant increase in vitamin B_6_ content and enhances the plant’s tolerance to various abiotic stresses (Bagri et al., 2018). In *Arabidopsis*, overexpression of both *AtPDX1.1* and *AtPDX2* has pleiotropic effects, including increased plant size, seed enlargement and increased weight, and taller plants. The excessive accumulation of vitamin B_6_ in these transgenic plants also confers increased resistance to oxidative stress (Raschke et al., 2011). Similarly, in tomato, Zhang et al. identified four genes associated with the vitamin B_6_ biosynthetic pathway, including *SiPDX1.2*, *SiPDX1.3*, and *SiPDX2*, which are involved in the *de novo* synthesis pathway, and *SiSOS4*, which is involved in the salvage pathway. By using the VIGS (Virus-Induced Gene Silencing) method, they investigated the role of vitamin B_6_ in tomato resistance to gray mold disease. The results showed that silencing the *SiPDX1.2* or *SiPDX1.3* reduced the resistance of tomato plants to gray mold. Further studies revealed that after infection by gray mold, the levels of vitamin B_6_ induced by the fungus decreased in the *SiPDX1.2* or *SiPDX1.3*-silenced plants (Zhang et al., 2014). This finding highlights the importance of vitamin B_6_ in plant defense mechanisms and suggests that genetic engineering could be a promising approach to improve plant resistance to diseases by modulating vitamin B_6_ levels.

To date, the molecular functions of genes involved in vitamin B_6_ biosynthesis, such as *Pdx1*, *Pdx2*, *Pdx3*, and *Sos4*, have been reported in several plants, including monocots like maize (Yang et al., 2017) and rice (Mangel et al., 2019), as well as dicots like arabidopsis (Raschke et al., 2011), tomato (Zhang et al., 2014), potato (Bagri et al., 2018), and cassava (Li et al., 2015). However, the functional role of the *NtPDX2* in tobacco remains unexplored. Therefore, in this study, we cloned the coding region sequence of *NtPDX2*, and used bioinformatics analysis to predict the phylogenetic relationship and driving function of this gene. Subcellular localization analysis and tissue expression profile analysis were used to reveal the biological functions of *NtPDX2* in different tissues. Overexpression and knockout of *NtPDX2* were studied to analyze the function of *NtPDX2* in regulating tobacco plant height and stem thickness. The molecular mechanism of *NtPDX2* in tobacco growth and development was analyzed by combining transcriptome sequencing technology and molecular biology methods to provide new gene resources and theoretical basis for breeding new tobacco varieties with good flue-cured tobacco quality. It also helps people to understand the functional scope of *PDXs* in a more comprehensive and detailed way and broaden the research field of *PDXs*.

## 2 Materials and methods

### 2.1 Identification and physicochemical property analysis of the *NtPDXs* family members in Nicotiana *tabacum* L.

In this study, we conducted a search based on the conserved domains of the PDX protein family, retrieving the conserved domain files for the glutaminase PdxT/SNO family and the PdxS/SNZ family from the Pfam database (http://pfam.xfam.org/). Subsequently, we utilized HMMER 3.0 software to screen for PDXs proteins in the tobacco genome database (https://solgenomics.net/organism/Nicotiana_tabacum/genome), using the aforementioned two conserved domains as references. Additionally, we searched for tobacco *PDXs* genes or proteins in the NCBI gene and genome databases (https://www.ncbi.nlm.nih.gov/) and the tobacco genome database using “pyridoxal 5’-phosphate synthase” as the keyword. Finally, we confirmed all identified *NtPDXs* family members through the CDD program on the NCBI website (https://www.ncbi.nlm.nih.gov/cdd/).

To predict the physicochemical properties of the *Pdx* gene family members, such as amino acid composition, molecular weight (MW), and isoelectric point (PI), the online tool ProtParam (http://web.expasy.org/protparam/) was utilized. Subcellular localization of the PDX was predicted using the Plant-mPLoc online tool (http://www.csbio.sjtu.edu.cn/bioinf/plant-multi/) and Euk-mPLoc 2.0 (http://www.csbio.sjtu.edu.cn/bioinf/euk-multi-2/) (Chou and Shen, 2010; Zhu et al., 2022).

### 2.2 Phylogenetic analysis of *Pdxs* family members

The gene IDs of *Pdxs* family members in maize (Yang et al., 2017), rice (Mangel et al., 2019), arabidopsis (Raschke et al., 2011), tomato (Zhang et al., 2014), potato (Bagri et al., 2018), and cassava (Li et al., 2015) were obtained from published studies. The gene and protein sequences of tobacco *Pdxs* were retrieved from their respective genome databases. The protein sequences of *Pdxs* from arabidopsis, maize, rice, tomato, potato, and cassava were aligned using MUSCLE software, and a phylogenetic tree was constructed using the Neighbor-Joining method. Bootstrap analysis with 1,000 replicates was conducted using MEGA-11 software to analyze the evolutionary relationships of *Pdxs* members across seven species (Kumar et al., 2018).

### 2.3 Protein-Protein interaction analysis between NtPDX1s and NtPDX2

Primers were designed to amplify the coding sequence of *NtPDX1.2* and *NtPDX1.3* genes without stop codons, as well as the complete coding sequence of *NtPDX2*, using *Nicotiana tabacum* L. leaf cDNA as a template via PCR. The PCR reaction conditions were as follows: initial denaturation at 95°C for 5 min, denaturation at 95°C for 30 sec, annealing at 56°C for 30 sec, extension at 72°C for 2 min, with a total of 30 cycles; followed by a final extension at 72°C for 10 min. The vector pCAMBIA1300-nLUC was used as the backbone, and the *NtPDX1.2* and *NtPDX1.3* coding sequences without stop codons were fused with the N-terminal gene of luciferase using the restriction enzymes *Bam*HI and *Sal*I, respectively. The constructs were then transformed into competent *Escherichia coli* to obtain the fusion expression vectors NtPDX1.2-nLUC and NtPDX1.3-nLUC. The vector pCAMBIA1300-cLUC was used as the backbone, and the *NtPDX2* coding sequence was fused with the C-terminal gene of luciferase using the restriction enzymes *Bam*HI and *Pst*I, resulting in the cLUC-NtPDX2 plant expression vector.

The constructed fusion expression vectors were transformed into Agrobacterium and injected into the leaves of 3-week-old *Nicotiana benthamiana*. After 24 hours of dark incubation and 40 to 48 hours of light incubation, the leaves were observed using a plant imaging system (NightShade LB985, Germany).

### 2.4 Subcellular localization analysis of NtPDX2

A plant expression vector was constructed by fusing the *NtPDX2* with the *eGFP*. The amplification primers were designed to exclude the stop codon, and cDNA from *Nicotiana tabacum* L. leaves was used as a template for PCR amplification to obtain the CDS fragment of *NtPDX2* without a stop codon. PCR conditions were as follows: 95°C for 5 min (initial denaturation), 95°C for 30 sec (denaturation), 56°C for 30 sec (annealing), 72°C for 2 min (extension), 30 cycles, and a final extension at 72°C for 10 min. The fusion expression vector was constructed using the pBI221 vector backbone, and restriction digestion with XbaI and SalI was performed on both the vector backbone and the PCR product. T4 ligase was used to ligate the vector and insert, which was then transformed into competent *Escherichia coli* to obtain the fusion expression vector pBI221-NtPDX2.

*Nicotiana tabacum* L. leaves at the 5-6 leaf stage were selected, and the top 2 leaves were cut into 1 mm wide strips. Approximately 20 leaf pieces were used to prepare protoplasts via enzymatic digestion. The fusion expression vector pBI221-NtPDX2 was transfected into tobacco protoplasts via PEG-mediated transformation. Localization was observed under a confocal microscope (Axio LSM 980, Zeiss Co., Ltd., Jena, Germany).

### 2.5 qRT-PCR analysis of *NtPDX2* expression in *Nicotiana tabacum* L. tissues

Total RNA was extracted from the roots, stems, and leaves of *Nicotiana tabacum* L. at different growth stages (seedling, rosette, and flower) using the RNAprep Pure Plant Plus kit (Tiangen, Beijing, China) following the manufacturer’s protocol. RNA quality and quantity were assessed by agarose gel electrophoresis and UV-spectrophotometry (BioPhotometer Plus, Eppendorf, Germany). First-strand cDNA was synthesized using the PrimeScript II First Strand cDNA Synthesis Kit (TaKaRa, Dalian, China). Real-time PCR was performed using the TB Green Premix Ex Taq™ (TaKaRa, Dalian, China). The reaction mixture (20 μL) included 10 μL of 2× TB Green Premix Ex Taq, 0.5 μL of forward and reverse primers (10 μM), 0.4 μL of 50× ROX reference dye II, 2.0 μL of cDNA template, and ddH2O. The reactions were conducted in an ABI7500 Real-Time PCR system (Applied Biosystems, Foster City, CA, USA). The *NtEF1α* was used as an internal reference (Schmidt and Delaney, 2010). The qPCR conditions were: 95°C for 30 sec, followed by 40 cycles of 95°C for 5 sec and 60°C for 34 sec. Data were analyzed using the 2^-ΔΔCt^ method with three biological replicates, and Pearson correlation analysis was performed to assess the qRT-PCR results.

### 2.6 Gene editing and overexpression vector construction, and positive plant identification

Gene editing vector construction: The CRISPR-P 2.0 online tool (http://crispr.hzau.edu.cn/cgi-bin/CRISPR2/CRISPR) was used to design sgRNA target sequences for the *NtPDX2*. The selected sgRNA sequences were synthesized by a commercial company, including the sgRNA guide sequence and gRNA scaffold. The *Xba*I and *Hin*dIII restriction sites were added to the 5’ and 3’ ends, respectively. The pCrispr-Cas9 vector and sgRNA-scaffold fragment were digested with *Xba*I and *Hin*dIII, and the resulting vector backbone and insert were ligated using T4 ligase (TaKaRa, Dalian, China) to generate the gene editing vector pCrispr-Cas9-sgRNA.

Overexpression Vector Construction: Primers were designed to amplify the full-length CDS of *NtPDX2*, with *Bam*HI and *Sac*I restriction sites added to the 5’ and 3’ ends, respectively. *Nicotiana tabacum* L. leaf cDNA was used as the template, and PCR amplification was performed under the following conditions: 95°C for 5 min (initial denaturation), 95°C for 30 sec (denaturation), 56°C for 30 sec (annealing), 72°C for 1 min (extension), 30 cycles, and a final extension at 72°C for 10 min. The pCambia2301 vector backbone was digested with *Bam*HI and *Sac*I, and the insert was ligated using T4 ligase, resulting in the overexpression vector pC23-35S2-PDX2-NOS.

Agrobacterium-mediated transformation was performed to introduce the constructs into *Nicotiana tabacum* L. plants (Niedbała et al., 2021). Transgenic plants were selected using kanamycin resistance and PCR analysis. All tobacco plants were grown in a greenhouse at 28°C with a 16-hour light/8-hour dark photoperiod and 50-65% relative humidity.

### 2.7 Measurement of plant height, stem thickness and vitamin B_6_ content

For T2 generation plants, 30 healthy and uniform overexpression, knockout, and wild-type plants were selected at maturity. Plant height, topping height, and stem girth were measured, and the data were analyzed for statistical significance using ANOVA and mean value calculation.

Stem tissues at the same position during the rosette stage were collected from overexpressing, knockout, and control plant lines, rapidly frozen in liquid nitrogen, and stored at -80°C. The determination of vitamin B_6_ content in the samples was carried out in accordance with the Chinese National Standard GB5009.154-2023. The standard reference materials used in the assay were purchased from Aladdin based on the CAS number.

### 2.8 RNA extraction, cDNA library construction, and transcriptome sequencing

Total RNA from each sample was extracted using the RNAprep Pure Plant Kit (Polysaccharide and Polyphenol-rich) (Tiangen, Beijing, China) according to the manufacturer’s protocol. A total of 27 samples from three biological replicates were collected. RNA quality was assessed by electrophoresis in a 1% agarose gel to check for genomic DNA contamination and degradation, and further quantified using a Kaiao K5500 spectrophotometer (Kaiao, Beijing, China) and an Agilent 2100 Bioanalyzer (Agilent Technologies, California, USA). After ensuring RNA quality, mRNA was enriched using magnetic beads with Oligo(dT) tails. The first strand of cDNA is then synthesized using a six-base random primer, and the second strand of cDNA is synthesized by adding buffer, dNTPs, RNase H, and DNA polymerase I (NEB, Massachusetts, USA). It was purified using QIAQuick PCR kit and eluted with EB buffer (QIAGEN, Germany). End repair, A-tailing, and adapter ligation were performed, followed by PCR amplification. The libraries were sequenced on the Illumina HiSeq 2000 platform.

### 2.9 Bioinformatics analysis of RNA-seq data to identify differentially expressed genes

The raw sequencing data were processed using CASAVA (v1.8) software for base calling and conversion. Sequence quality was assessed by examining base sequencing error rates and GC content distribution. After filtering out adapter sequences, reads with more than 5% N content, and low-quality reads, high-quality clean reads were obtained. In this study, the whole genome data of tobacco was used as the reference genome sequence (https://solgenomics.net/ftp/genomes/Nicotiana_tabacum/edwards_et_al_2017/assembly/), and the TopHat2 software to analyze the genomic localization of clean reads from stem tissues of different tobacco plants. StringTie software was used to reassemble all clean reads to predict new transcripts, and gene expression levels were quantified by FPKM using HTSeq software. DEGseq software was used to identify differentially expressed genes (DEGs) based on a threshold of p-value < 0.05 and |log_2_ (Fold Change)| > 2.0. Finally, statistics on differentially expressed genes between different tissues were derived.

### 2.10 Functional annotation of differentially expressed genes

The DEGs were subjected to Gene Ontology (GO) and Kyoto Encyclopedia of Genes and Genomes (KEGG) pathway enrichment analysis. GO enrichment was performed using the GOseq R package (v4.0.2), and KEGG pathway analysis was conducted using KOBAS (v3.0). GO terms and KEGG pathways with a corrected p-value < 0.05 were considered significantly enriched. Based on these results, the main functions and metabolic pathways of the DEGs were hypothesized.

### 2.11 qRT-PCR validation of RNA-seq results

To validate the accuracy of differential gene expression patterns related to plant height, 15 significantly differentially expressed genes were selected for qRT-PCR verification. Specific primers were designed using Primer3 (http://bioinfo.ut.ee/primer3-0.4.0/) (see **Supplementary Table S5**). cDNA was synthesized from 1 μg of total RNA using the PrimerScript RT Kit (TAKARA, Dalian, China) according to the manufacturer’s instructions, with a 20 μL reaction mixture. qRT-PCR was performed using SYBR Premix Ex Taq II (TAKARA, Dalian, China), with the tobacco *NtEF1α* as the internal control. The PCR conditions were 95°C for 30 sec, followed by 40 cycles of 95°C for 5 sec and 60°C for 34 sec. Three biological replicates and technical replicates were performed, and the relative gene expression levels were quantified using the 2^-ΔΔCt^ method.

## 3 Results

### 3.1 Identification and phylogenetic analysis of tobacco *Pdxs* family members

Initially, we performed BLAST searches using *Pdxs* from previously studied species to identify five potential *NtPDXs* sequences within the tobacco genome database. Subsequently, we conducted conservation domain analysis and phylogenetic analysis to ascertain whether these genes are indeed part of the target gene family. If these genes possess the typical conserved domains and cluster with other known family members in the phylogenetic tree will be considered as candidate genes. Finally, a total of three NtPDXs protein sequences were obtained. These were aligned with *Pdxs* from *Arabidopsis thaliana* using BLAST, and named accordingly as *NtPDX1.2*, *NtPDX1.3*, and *NtPDX2* (CDS sequences and their encoded protein sequences are provided in **Supplementary File S1**).

Analysis of the sequence information of the *NtPDXs* family members revealed that the CDS lengths of the *NtPDX1.2*, *NtPDX1.3*, and *NtPDX2* are 921, 930, and 756 bp, respectively. The *NtPDX1.2* is intron-less, the *NtPDX1.3* contains one intron, and the *NtPDX2* contains six exons (**Supplementary Figure S1**). The encoded amino acids are 306, 309, and 251 aa, with protein molecular weights of 32.76, 33.09, and 27.15 kDa (**Table 1**). Utilizing bioinformatics analysis software or websites such as ProtParam and Plant-mPLoc to predict the physicochemical properties and subcellular localization of the NtPDXs family proteins, it was found that the theoretical pIs of NtPDX1.2, NtPDX1.3, and NtPDX2 proteins are 5.01, 5.92, and 5.31, respectively. All proteins are hydrophilic, lack signal peptides and transmembrane domains, and subcellular localization predictions indicate that they are all localized to the cytoplasm (**Table 1**).

**Table 1:** Analysis of basic physicochemical properties of tobacco NtPDXs proteins.

Physicochemical Properties	NtPDX1.2	NtPDX1.3	NtPDX2
Isoelectric Point (pI)	5.01	5.92	5.31
Molecular Weight (kDa)	32.76	33.09	27.15
Hydrophobicity/Hydrophilicity	Hydrophilic	Hydrophilic	Hydrophilic
Transmembrane domain	No	No	No
Signal Peptide	No	No	No
Subcellular Localization	Cytoplasm	Cytoplasm	Cytoplasm

**Figure 1:**
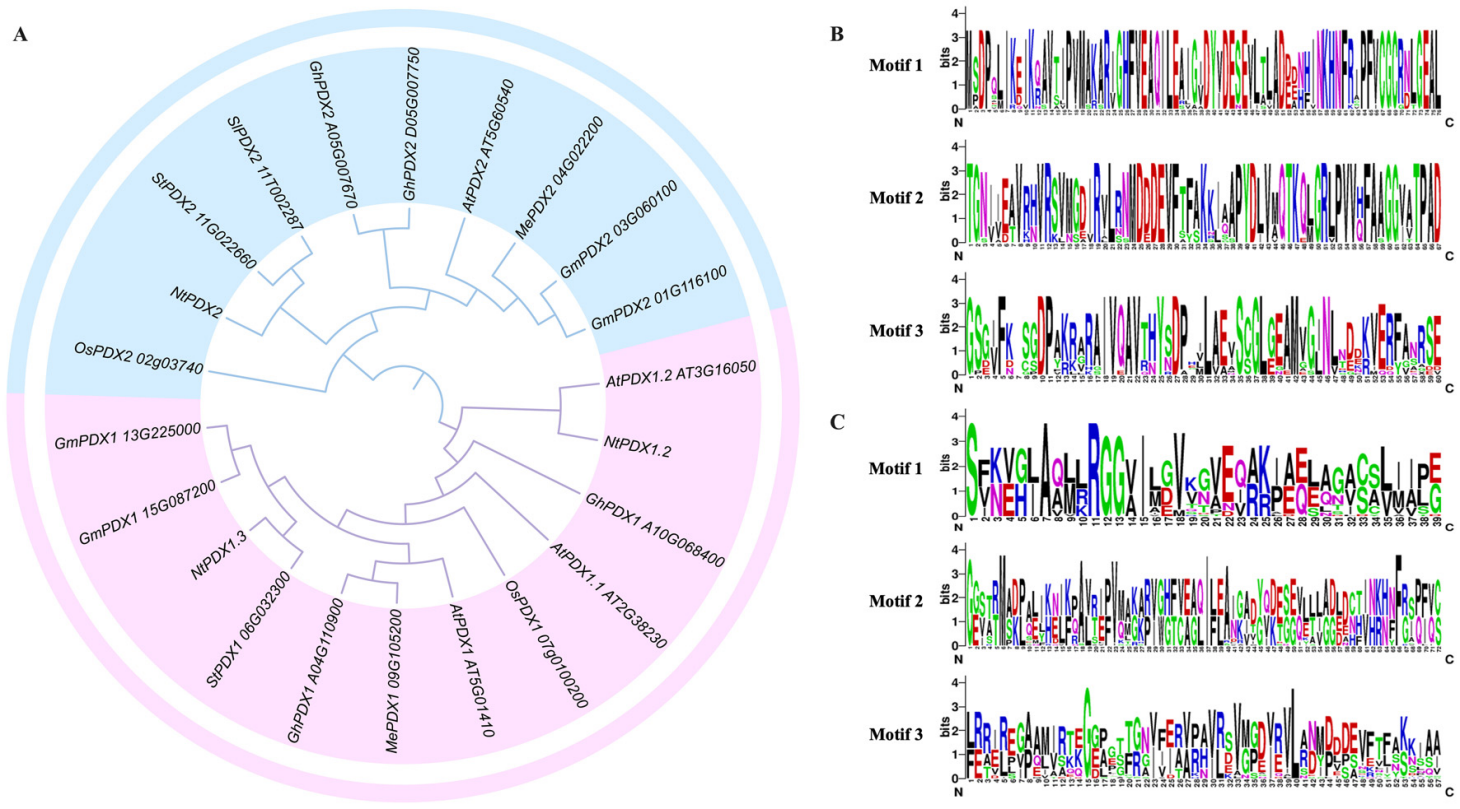
Phylogenetic relationships of PDXs members from different species and their motif analyses. **(A)** unrooted phylogenetic tree was constructed using MEGA-11 by neighbor-joining method and 1,000 bootstrap replicates. A total of 22 PDXs members were categorized into two subfamilies: distinguished by light blue and light red folds, respectively. **(B)** motif analysis of PDX1s family members; **(C)** motif analysis of PDX2s family members.

The amino acid sequences of 22 PDXs from Arabidopsis, maize, rice, tomato, potato, cassava, and tobacco were aligned using the ClustalW software, and their phylogenetic relationships were analyzed using MEGA-11 software. The analysis indicated that all PDXs members can be distinctly divided into two groups, with the first group being the PDX1 clade and the second group being the PDX2 clade (**Figure 1A**). This suggests that the biological functions of PDX1 and PDX2 are significantly different and can be completely separated. Through the analysis of the conserved sequences of the PDX2s protein family, it was found that this family of proteins contains three relatively conserved motifs, with the catalytic active centers located within these three motifs (**Figures 1B, C**).

### 3.2 Analysis of protein-protein interaction between *NtPDX1* and *NtPDX2*

**Figure 2:**
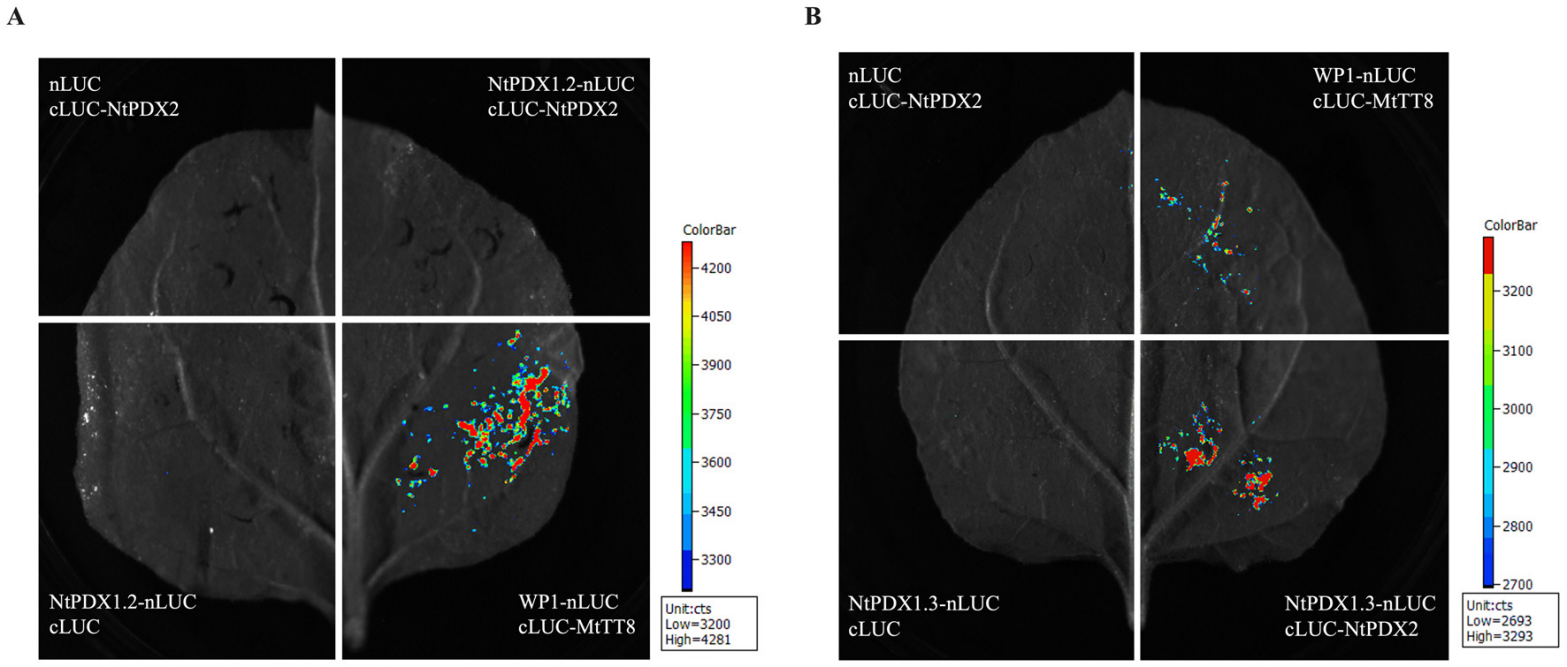
Split-LUC experiment verifies the protein interaction between *NtPDX1.2*, *NtPDX1.3* and *NtPDX2.* **(A)** Interaction between *NtPDX1.2* and *NtPDX2*; **(B)** Interaction between *NtPDX1.3* and *NtPDX2*. WP1-nLUC and cLUC-MtTT8 was used as a positive control (Meng et al., 2019).

In this study, we used the luciferase complementation (Split-LUC) assay to verify whether *NtPDX1.2* and *NtPDX1.3* were able to complement with *NtPDX2* to form a complex. The results showed that *NtPDX1.2* and *NtPDX2* were unable to detect fluorescent signals, whereas *NtPDX1.3* and *NtPDX2* were able to undergo complementation to form a complex and detect fluorescent signals (**Figure 2**). It indicates that *NtPDX1.3* and *NtPDX2* are able to form a functional complex in tobacco to catalyze the synthesis of PLP.

### 3.3 Subcellular localization analysis using PEG-mediated transfection

**Figure 3:**
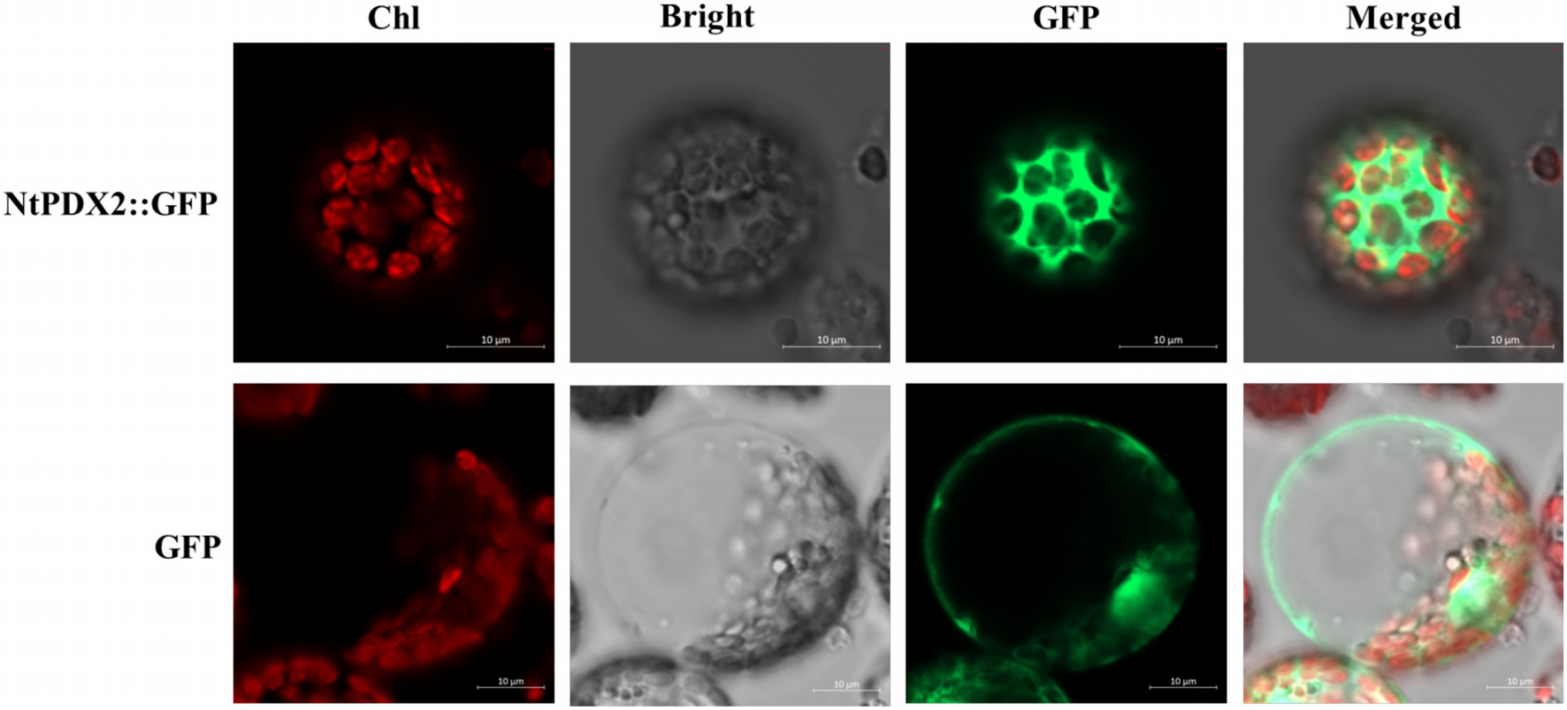
Subcellular localization of NtPDX2 in tobacco protoplasts. Red (Chl) indicates chloroplast autofluorescence, grey (Bright) shows bright field, green (GFP) indicates green fluorescence signal, and the merged image (Merged) shows the image rendered by merging the three channels.

To ascertain the subcellular localization of the NtPDX2 protein in tobacco cells, a transient expression assay was conducted using the PEG transfection method. Tobacco protoplasts were transfected with the pBI221-eGFP empty vector and the pBI221-NtPDX2 fusion expression vector. Fluorescence imaging was then performed using a laser confocal microscope to observe the expression patterns. It was found that the eGFP from the empty vector was expressed to some extent in the nucleus, cell membrane, and cytoplasm. In contrast, the pBI221-NtPDX2 fusion expression vector exhibited green fluorescence signals exclusively in the cytoplasm, with no observable signals elsewhere (**Figure 3**). This result indicates that the *NtPDX2* gene functions within the cytoplasm of the cells.

### 3.4 Tobacco genetic transformation and positive plant identification

Genetic transformation of tobacco was performed using the Agrobacterium-mediated method. *Nicotiana tabacum* L. leaves were inoculated with *Agrobacterium*, followed by callus induction, selective screening of resistant calli, formation of embryogenic callus, development of adventitious bud, rooting, acclimatization, and soil cultivation (**Supplementary Figure S2A**). A total of 28 kanamycin-resistant *Nicotiana tabacum* L. T0 generation plants and 16 gene-edited *Nicotiana tabacum* L. T0 generation plants were obtained. Genomic DNA was extracted from all regenerated *Nicotiana tabacum* L. plants, and PCR amplification followed by Sanger sequencing was used for identification (**Supplementary Figure S2B**). This analysis identified 22 plants with successful overexpression and 12 gene-edited plants.

In the gene-edited plants, sequencing revealed an insertion of a “T” nucleotide at the third base after the sgRNA target site, distal to the PAM sequence (5’-TGG-3’) (**Supplementary Figure S2C**). This insertion caused a frameshift mutation in the encoded protein, leading to a premature stop codon, which interrupted translation and resulted in the inability to produce a functional NtPDX2 protein.

### 3.5 Analysis of *NtPDX2* expression levels and vitamin B_6_ content in overexpressing and knockout plants

**Figure 4:**
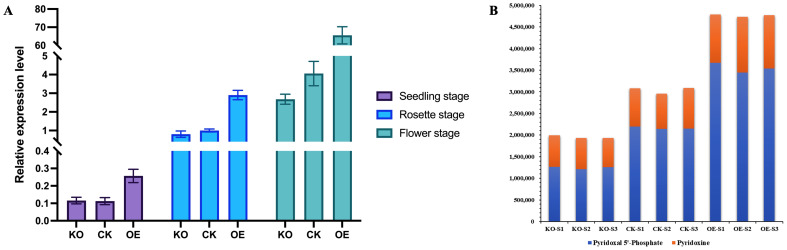
Analysis of *NtPDX2* gene expression level and determination of vitamin B_6_ content in overexpression plants and knockout plants. **(A)** Analysis of *NtPDX2* gene expression levels in overexpression and knockout plants, purple indicates seedling stage, blue indicates rosette stage, and green indicates flowering stage. All data were quantitatively analyzed as the expression of *NtPDX2* in stem tissues of recipient tobacco at the rosette stage. **(B)** Analysis of *NtPDX2* expression level and determination of vitamin B_6_ content in overexpression plants and knockout plants, Units are mAU*min orange indicates Pyridoxine content and blue indicates Pyridoxal 5’-Phosphate content. KO represents knockout plants, CK represents recipient plants, and OE represents overexpressed plants.

To determine whether the expression level of the *NtPDX2* changes in overexpressing and knockout plants, qRT-PCR was used to analyze the expression levels in stem tissues of overexpressing, knockout, and wild-type control plants at different growth stages. The results showed that within the same plant line, the expression level of *NtPDX2* in stem tissues was highest during flowering, followed by the rosette stage, with the lowest expression observed at the seedling stage. In comparison to the control plants, the expression level of *NtPDX2* was significantly increased in the overexpressing plants, with a 3- to 15-fold higher expression. There was no significant difference in the expression level between the knockout plants and the control plants (**Figure 4A**). These findings suggest that the expression of *NtPDX2* is notably upregulated during the stem elongation period in tobacco.

Subsequently, liquid chromatography-tandem mass spectrometry (LC-MS) was used to measure the vitamin B_6_ content in stem tissues during the rosette stage for the overexpressing, knockout, and control plant lines. The results showed that the vitamin B_6_ content was highest in the stem tissues of the overexpressing plants, reaching 1.5 times that of the recipient plants, while it was lowest in the knockout plants, at two-thirds the level of the recipient plants (**Figure 4B**).

### 3.6 Measurement and statistical analysis of plant height and stem thickness in tobacco

A total of 30 plants from each of the T2 generation overexpressing, knockout, and wild-type control lines were selected, and plant height and stem girth were measured at different developmental stages (seedling, rosette, and flowering stages). The results showed that at the seedling stage, the overexpressing plants were the tallest compared to the control plants, while the knockout plants did not show significant differences from the control plants (**Supplementary Figure S3A**). During this early growth stage, stem tissues had not yet undergone significant growth, so measurable data could not be obtained.

**Figure 5:**
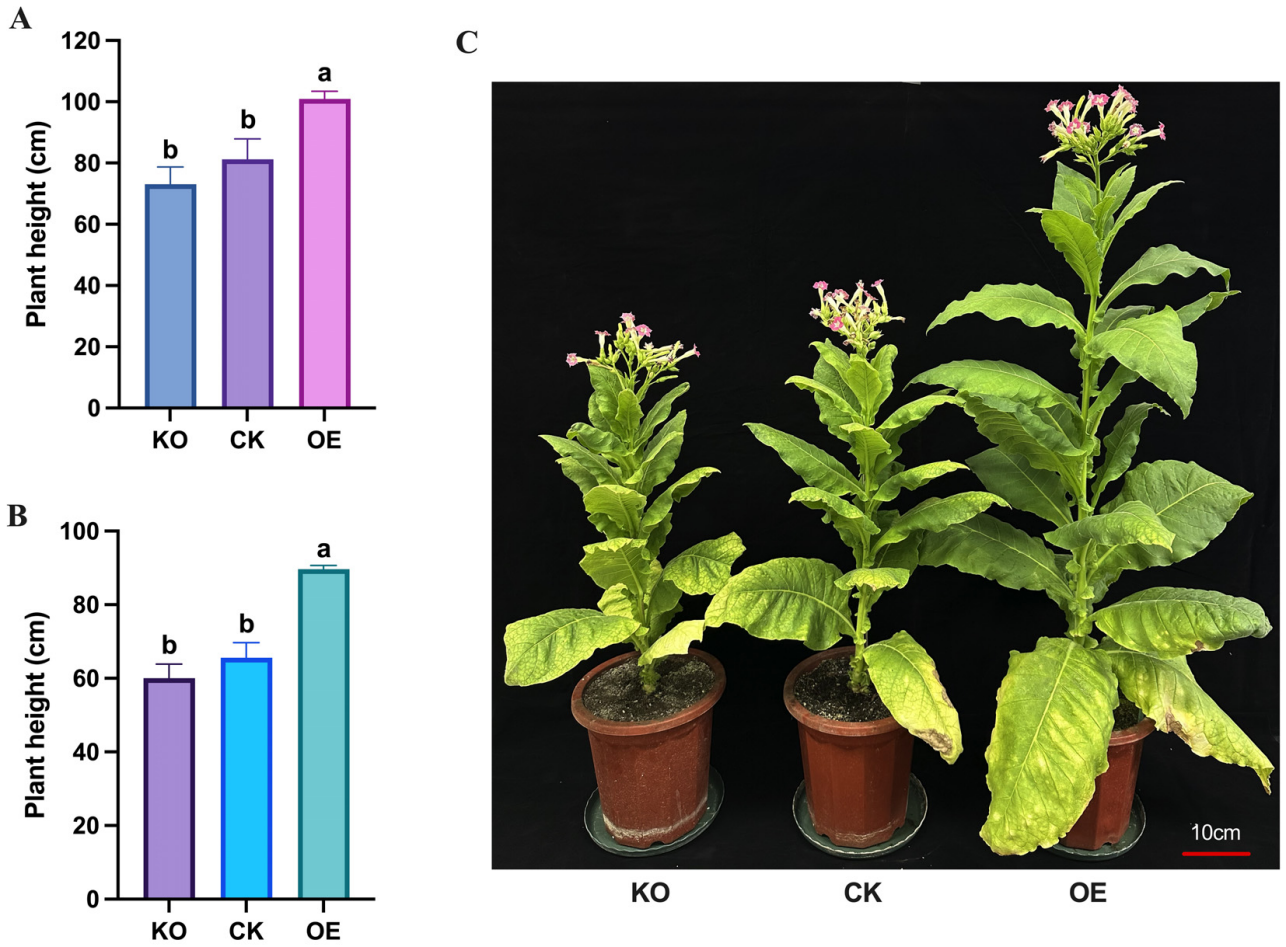
Measurement and statistical analysis of plant height traits of tobacco plants at flowering stage. **(A)** Measurement data of total height of different plants at flowering stage. **(B)** Measurement data of topping height of different plants at flowering stage. **(C)** Comparison of plant height of different plants at flowering stage. KO represents knockout plants, CK represents recipient plants, and OE represents overexpressed plants. Lowercase letters (a, b, c) indicate significance at α = 0.05.

At the rosette stage, the overexpressing plants had a significantly taller plant height compared to both the knockout and control plants. The knockout plants did not differ significantly from the control plants in terms of height (**Supplementary Figure S3B**). However, at the flowering stage, the overexpressing plants had a significantly greater plant height (100.93 ± 2.55 cm) compared to the control plants (81.20 ± 6.70 cm), while the knockout plants (73.10 ± 5.62 cm) were considerably shorter than the control plants (**Figures 5A, C**). Similarly, the topping plant height of overexpressing tobacco plants (89.63 ± 1.03 cm) was significantly higher than that of control plants (65.57 ± 4.15 cm), and the topping plant height of knockout plants (60.03 ± 3.81 cm) was significantly lower than that of the control plants (**Figure 5B**).

**Figure 6:**
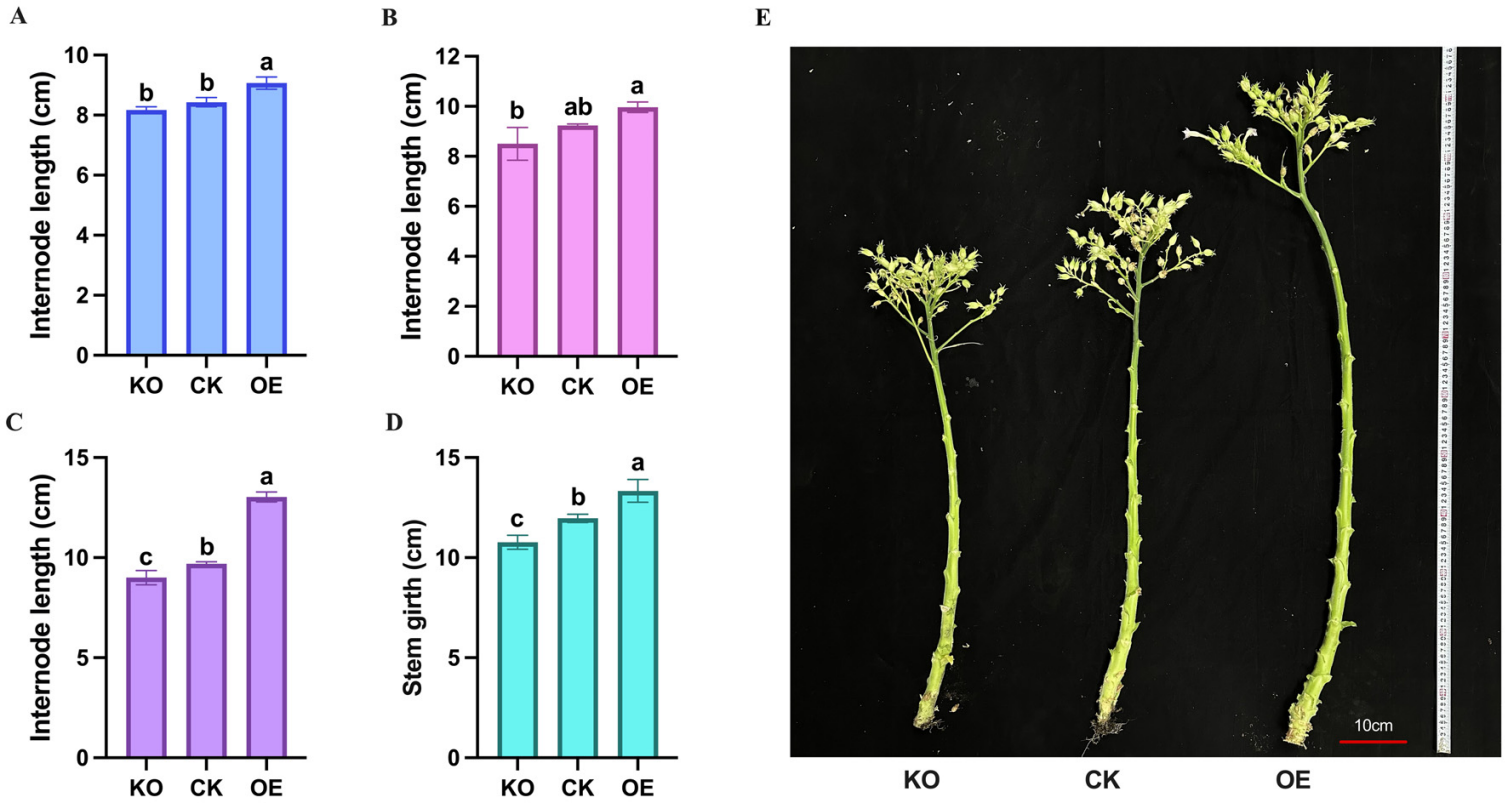
Measurement and statistical analysis of stem internode length and girth of tobacco plants. **(A)** Measurement data of bottom internode length of different plants at flowering stage. **(B)** Measurement data of middle internode length of different plants at flowering stage. **(C)** Measurement data of top internode length of different plants at flowering stage. **(D)** Measurement data of stem girth of different plants at flowering stage. **(E)** Comparison of stem phenotypes of different plants at flowering stage. KO represents knockout plants, CK represents recipient plants, and OE represents overexpressed plants. Lowercase letters (a, b, c) indicate significance at α = 0.05.

Subsequently, internode lengths were measured at different positions along the stem, with the total length of three adjacent internodes used as a single data point. The results showed that the upper internodes grew the longest, followed by the middle internodes, while the lower internodes were shorter and more densely packed. Statistically, the overexpressing plants had longer internodes at all positions compared to the control and knockout plants, with the knockout plants having the shortest internodes (**Figures 6A–C**).

For stem girth measurements, the overexpressing plants had the thickest stems (13.64 ± 0.35 cm) at the same position, followed by the control plants (12.02 ± 0.21 cm), and the knockout plants had the thinnest stems (10.83 ± 0.24 cm) (**Figures 6D, E**). These results indicate that the *NtPDX2* gene plays a positive regulatory role in promoting both stem elongation and thickening in tobacco.

### 3.7 Transcriptomic data analysis of tobacco stem tissue at the rosette stage

High-throughput RNA sequencing was performed using the DNBSEQ-T7 platform on three biological replicates of *Nicotiana tabacum* L. stem tissue samples, with a total of 9 samples. The sequencing generated 41.08-44.84 million raw reads. After removing adapter sequences, low-quality reads, and raw reads with more than 5% N values, a total of 37.72-41.13 million clean reads (91.74%-93.10% of the raw reads) were obtained. Each sample group had three biological replicates, and the results indicated high consistency between RNA-seq data (0.918 < R² < 0.964).

For all sequencing data, Q20 and Q30 values were above 97.77% and 95.03%, respectively. The GC content ranged from 44.75% to 51.95%, and the error rate for all samples was between 0.01% and 0.02%. Overall, 92.82% to 97.05% of the high-quality clean reads were mapped to the tobacco reference genome using Hisat2 (v2.2.1) software (see **Table 2**). Gene expression distribution was analyzed for each tissue sample (see **Supplementary Table 3**), with the number of genes exhibiting high expression abundance ranging from 13,216 to 16,376. Among the mapped reads, 71.23%-94.39% were located in the exonic regions, 1.59%-4.23% in intronic regions, and 2.11%-16.60% in intergenic regions (see **Table 2**). These results indicate that the transcriptomic data generated by RNA sequencing are rich and reliable, with high accuracy.

**Table 2:** Statistical table of transcriptome sequencing data of stem tissues from different tobacco materials.

Sample	KO-S1	KO-S2	KO-S3	CK-S1	CK-S2	CK-S3	OE-S1	OE-S2	OE-S3
Raw Reads	41817600	41268232	41319018	41077414	41953984	44837684	43639280	43606482	41398914
Raw Bases	6272640000	6190234800	6197852700	6161612100	6293097600	6725652600	6545892000	6540972300	6209837100
Clean Reads	38801068	38411974	38003458	37719444	38603284	41132906	40626908	40456910	38038996
Clean Bases	5776246440	5720818237	5654762644	5617203402	5749999552	6125353982	6048443566	6022951074	5662500174
Clean read rate (%)	92.79%	93.08%	91.98%	91.83%	92.01%	91.74%	93.10%	92.78%	91.88%
Q20 bases rate (%)	97.84	97.95	97.82	98	98.05	97.9	98.17	98.14	97.77
Q30 bases rate (%)	95.29	95.46	95.21	95.49	95.58	95.26	95.88	95.69	95.03
GC content (%)	45.62	47.73	46.21	50.68	51.95	51.53	44.75	45.89	46.95
Mapped reads	36038431	36476010	35521832	36406807	37464487	39652121	37709896	38349104	35809910
Mapped rate (%)	92.88	94.96	93.47	96.52	97.05	96.4	92.82	94.79	94.14
Exon (%)	87.19%	89.63%	94.39%	71.23%	81.55%	85.72%	88.44%	77.87%	89.75%
Intron (%)	3.64%	3.12%	3.50%	2.17%	1.59%	1.69%	4.23%	3.66%	3.37%
Intergenic (%)	9.16%	7.25%	2.11%	26.60%	16.86%	12.59%	7.32%	18.47%	6.88%

**Figure 7:**
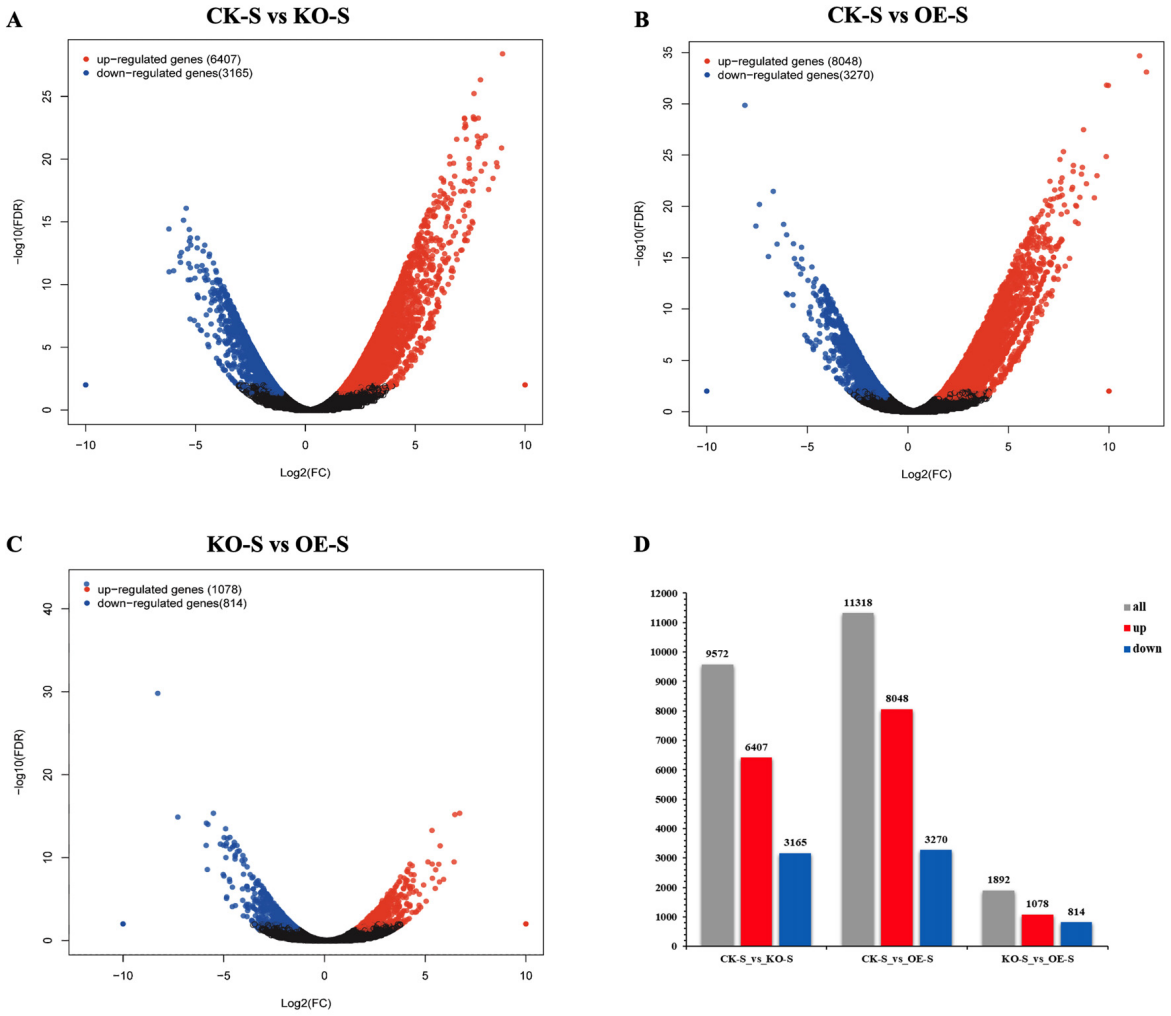
Up-regulation and down-regulation of differentially expressed genes (DEGs) in stem tissues of different tobacco materials. **(A–C)** Volcano map of differentially expressed genes (DEGs) (red dots represent up-regulated DEGs, blue dots represent down-regulated DEGs, black dots represent undifferentially expressed genes, horizontal coordinates represent multiples of gene expression in different samples, and vertical coordinates represent statistically significant differences in gene expression). **(D)** Comparison of up-regulated and down-regulated DEGs in each pair of samples between stem tissues of different tobacco materials (gray bar chart represents all DEGs, red bar chart represents up-regulated DEGs, blue bar chart represents down-regulated DEGs, horizontal coordinate represents the name of the sample comparison group, and vertical coordinate represents the number of DEGs).

Clustering and differential gene expression analyses were performed based on the FPKM values for each gene in different tissues. Volcano plots and histograms were generated to display the number of DEGs in each pairwise comparison (**Figure 7**). According to the sequencing results, volcano plots were created, and genes with significant differences in expression |log_2_ (Fold Change) | > 2 and FDR < 0.05. were selected. The volcano plots revealed numerous upregulated and downregulated genes in pairwise comparisons (**Figures 7A–C**). Histograms summarizing the significant DEGs identified in all pairwise comparisons across samples were also generated (**Figure 7D**).

Subsequently, the transcriptomic data from the pairwise comparisons were compared to gene annotation databases. A total of 4,438, 5,402, and 830 genes were matched in the Gene Ontology (GO) database, while 1,835, 2,166, and 322 genes were matched in the Kyoto Encyclopedia of Genes and Genomes (KEGG) database (**Table 3**).

**Table 3:** Summary of annotated genes in each database for the pairwise comparisons.

Pairwise Comparisons	GO	KEGG
CK-S vs KO-S	4438	1835
CK-S vs OE-S	5402	2166
KO-S vs OE-S	830	322

**Figure 8:**
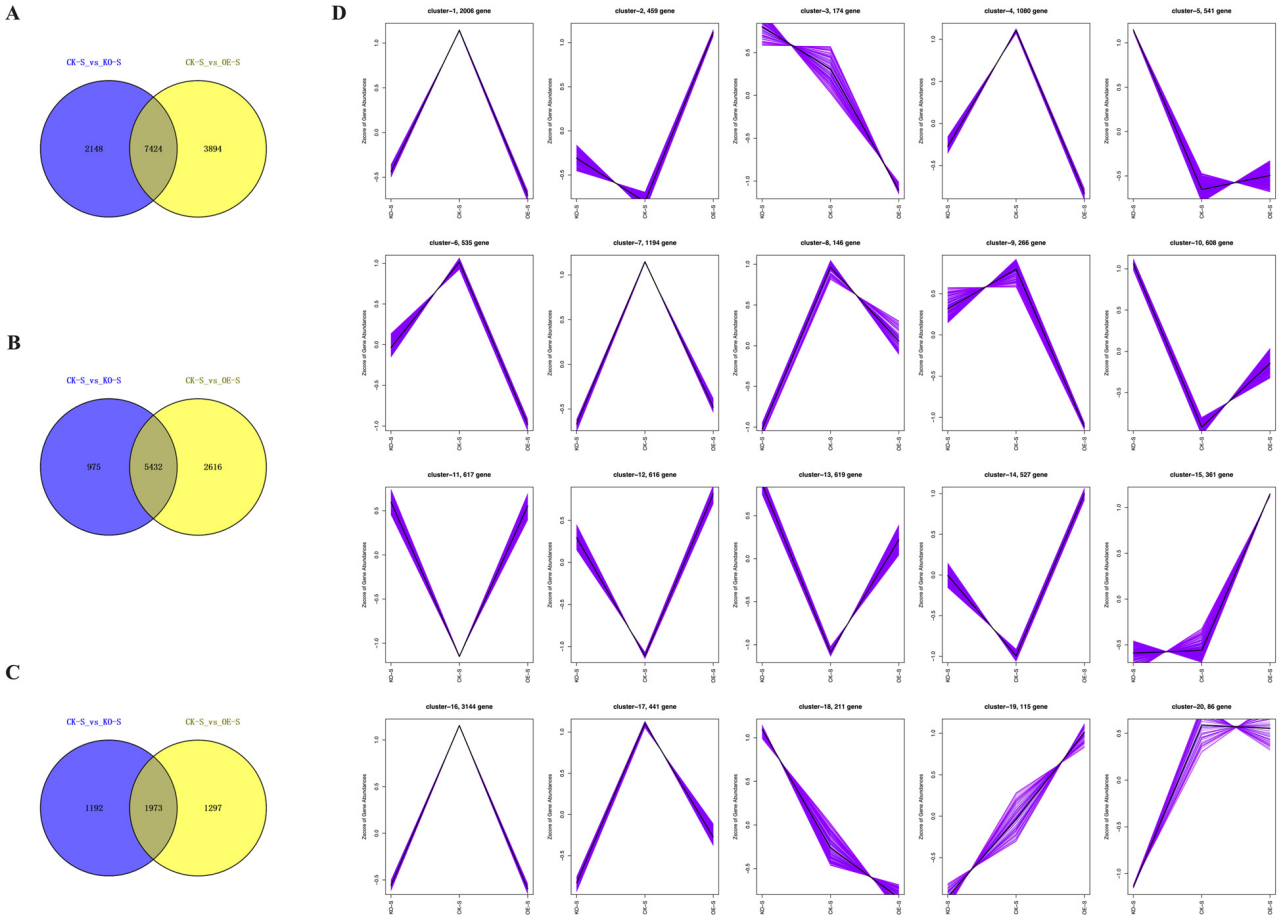
DEGs identified in comparison of stem tissues in different tobacco lines. **(A-C)** Venn diagram analysis of the all-DEGs, up-DEGs and down-DEGs in stem tissues of CK-S vs KO-S and CK-S vs OE-S. **(D)** K-mean clustering analysis on differentially expressed genes.

To identify genes that are specifically or predominantly expressed during the stem elongation phase, Venn diagrams were used to screen differentially expressed genes (DEGs) between the overexpressing, knockout, and wild-type control plants. Based on the transcriptomic data, K-meansclustering analysis was conducted to generate a trend graph. Additionally, Venn diagramswere constructed to illustrate the number of differentially expressed genes (DEGs) in eachpairwise comparison (**Figure 8**).

Comparative analysis of DEGs between stem tissues of CK-S vs KO-S and CK-S vs OE-S showed a total of 7,424 DEGs (**Figure 8A**). Of these, 5,432 DEGs were upregulated (**Figure 8B**), and 1,973 DEGs were downregulated (**Figure 8C**). Trend graphanalysis revealed changes in DEGs. Compared to the control plants, a large number of DEGs were upregulated in the overexpressing plants, while many were downregulated in the knockout plants. Conversely, several DEGs were downregulated in the overexpressing plants and upregulated in the knockout plants (**Figure 8D**).

The dynamic changes in DEGs identified through comparative transcriptomic analysis of different tobacco stem tissues may reveal the regulatory mechanisms of key genes involved in stem elongation and development.

### 3.8 Functional annotation of differentially expressed genes (DEGs) in tobacco stem tissue transcriptomic libraries

To identify genes associated with stem elongation and development, we analyzed the differential expression of genes in the stem tissue transcriptomic data between the overexpressing, knockout, and wild-type control plants. Comparisons between the control and knockout plants, control and overexpressing plants, and knockout and overexpressing plants identified 9,572, 11,318, and 1,892 DEGs, respectively. These DEGs were functionally annotated using Gene Ontology (GO) terms and Kyoto Encyclopedia of Genes and Genomes (KEGG) metabolic pathways.

**Figure 9:**
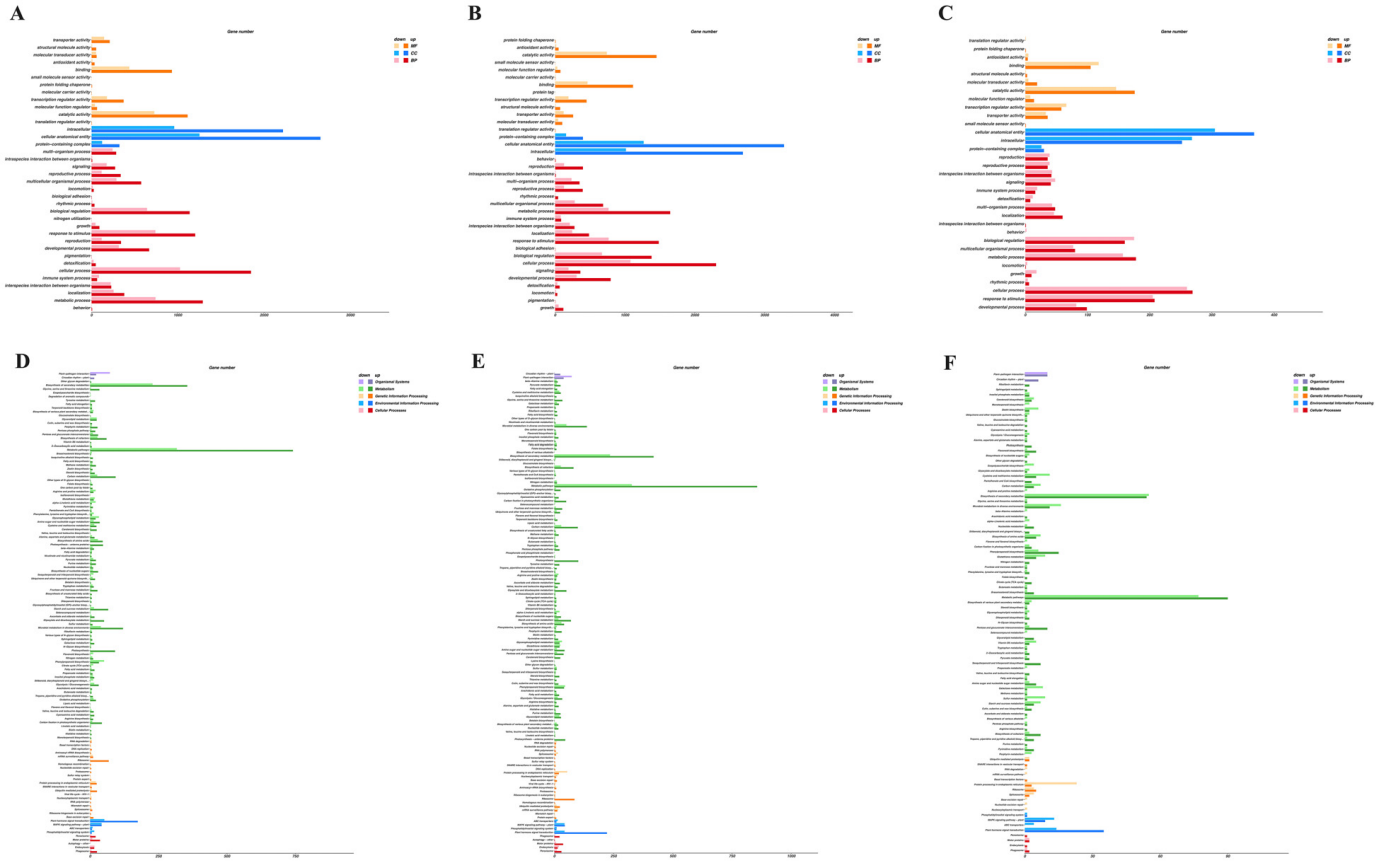
Functional annotation analysis of DEGs was conducted according to GO terms and KEGG metabolic pathway. **(A)** Enriched GO terms in CK-S vs. KO-S; **(B)** enriched GO terms in CK-S vs. OE-S; **(C)** enriched GO terms in KO-S vs. OE-S; **(D)** enriched KEGG metabolic pathways in CK-S vs. KO-S; **(E)** enriched KEGG metabolic pathways in CK-S vs. OE-S; **(F)** enriched KEGG metabolic pathways in KO-S vs. OE-S.

In the GO term analysis, a total of 4,438, 5,402, and 830 DEGs with functional annotations were enriched in 38, 38, and 34 GO categories, respectively, for the three pairwise comparisons (**Figures 9A–C**). In all three comparisons, DEGs related to biological processes were significantly enriched in processes such as cellular processes, metabolic processes, responses to stimuli, and biological regulation. In the cellular component category, DEGs were mainly distributed in functional groups related to cellular structures, intracellular components, and protein complexes. In terms of molecular functions, DEGs were primarily enriched in functional groups related to catalytic activity, binding, transcription factor activity, and transport activity. These results suggest that these functional groups may play important roles in stem elongation and development.

In the KEGG pathway analysis, a total of 1,835, 2,166, and 322 DEGs from the three comparisons were significantly enriched in 127, 129, and 99 metabolic pathways, respectively (**Figures 9D–F**). These pathways primarily involved metabolic pathways, biosynthesis of secondary metabolites, plant hormone signal transduction, plant-pathogen interactions, and carbon metabolism. These results indicate that these functional groups and metabolic pathways may play crucial roles during the stages of stem development and elongation.

### 3.9 Analysis of functionally related genes involved in stem tissue elongation and development

**Figure 10:**
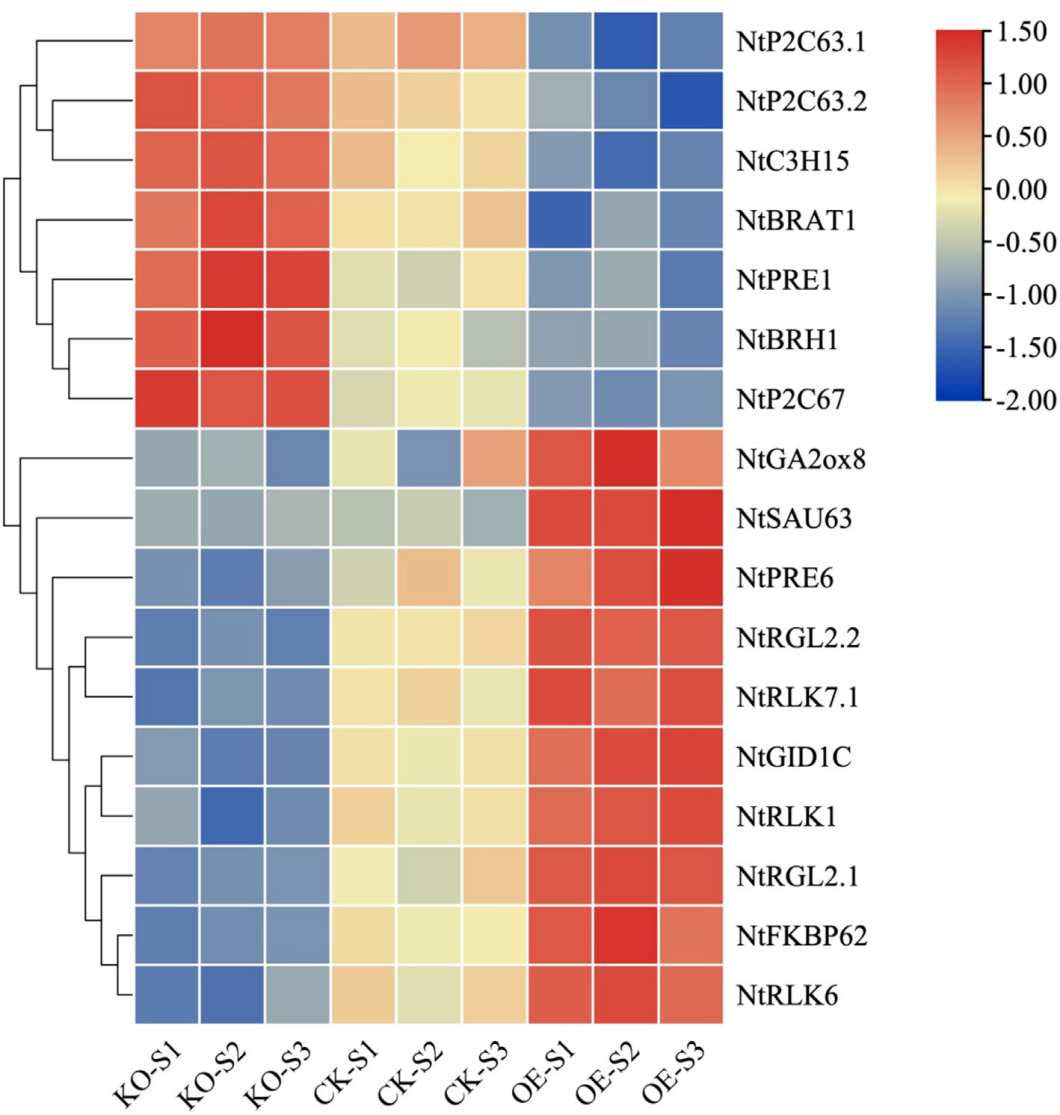
Heatmap analysis of related functional genes that have been reported to involve in the development of stem tissue elongation (P-value < 0.05, |log_2_ Fold Change| >2 at one sampling point).

During the developmental and elongation phases of stem tissue, metabolic activities within the stem cells are highly vigorous, particularly characterized by the overexpression of three categories of genes: 1) Genes associated with cellular structure or the cytoskeleton; 2) Genes related to transcription factors that regulate cell growth; 3) Genes associated with the biosynthesis of plant hormones. A selective analysis of functionally related genes that are differentially expressed during the elongation and development of stem tissue has been conducted, with reported gene expression patterns related to stem tissue elongation and development as shown in **Figure 10**. Numerous positive regulatory genes are involved in the elongation and development of stem tissue cells, such as BR signaling-related receptor kinases (*NtBRAT1*), gibberellin synthesis-related genes (*NtRGL2.1*, *NtRGL2.2*, *NtGID1C*, and *NtGA2ox8*), PRE subgroup transcription factors that integrate multiple plant hormones (*NtPRE1* and *NtPRE6*), lectin receptor kinases (*NtRLK1*, *NtRLK6*, *NtRLK7*), and FKBP-type isomerases related to protein folding (*NtFKBP62*). These genes are highly expressed in the overexpressing tobacco stem tissue and exhibit lower expression levels in the knockout tobacco plants compared to the recipient plants. Conversely, there are negative regulatory genes that participate in the elongation and development of stem tissue, such as RING finger proteins (*NtBRH1*), protein phosphatases (*NtP2C63.1*, *NtP2C63.2*, and *NtP2C67*), CCCH-type zinc finger proteins (NtC3H15), and SAUR-like auxin-responsive proteins (*NtSAU63*). These genes are highly expressed in the stem tissue of knockout tobacco plants and have the lowest expression levels in the overexpressing plants. These genes have been reported to regulate the elongation and development process of stem tissue positively or negatively.

## 4 Discussion

Vitamin B_6_, a water-soluble vitamin. It is essential for all living organisms in their life processes (Mangel et al., 2019). The primary metabolic active form of vitamin B_6_ is pyridoxal 5’-phosphate (PLP), which is synthesized *de novo* through the involvement of two glutamine transferases, PDX1 and PDX2. The *de novo* biosynthesis of PLP can only proceed when PDX1 and PDX2 form a functional complex, utilizing ribose 5-phosphate (R5P), glyceraldehyde 3-phosphate (G3P), and glutamine as substrates (Moccand et al., 2011). Among them, the PDX2 subunit acts as an ammonium donor, whereas PDX1 serves as an ammonium acceptor and also functions as a synthetase, which is responsible for synthesizing pyridoxal phosphate (PLP) by combining the provided ammonium group with other substrates.

In this study, we used the coding sequences (CDS) of the Arabidopsis *Pdxs* as references and performed a BLAST search in the tobacco genome database, identifying two *NtPDX1* (*NtPDX1.2* and *NtPDX1.3*) and one *NtPDX2*. Analysis of the physicochemical properties of the protein sequences revealed that the PDX proteins do not have signal peptides or transmembrane domains, and subcellular localization analysis showed that they are localized in the cytoplasm. These results are consistent with findings from other species, such as maize (Yang et al., 2017), rice (Mangel et al., 2019), and arabidopsis (Raschke et al., 2011). In *Arabidopsis*, there are three functional homologs of *PDX1* (*AtPDX1.1*, *AtPDX1.2*, and *AtPDX1.3*) and one homolog of *PDX2*. However, only *AtPDX1.1* or *AtPDX1.3* can form a functional complex with *AtPDX2* to catalyze the *de novo* synthesis of vitamin B_6_, *AtPDX1*.2 cannot form a functional complex with AtPDX2. In fact, both *AtPDX1* and *AtPD*X2 are essential for vitamin B_6_ biosynthesis in plants (Titiz et al., 2006). Overexpression of any B_6_ pathway gene in *Arabidopsis*, such as PDX1 or PDX2, results in increased expression at the mRNA level, and the overexpression lines accumulate excessive vitamin B_6_ (Chen and Xiong, 2009; Raschke et al., 2011). Subsequently, we verified the interactions between *NtPDX1.2*, *NtPDX1.3*, and *NtPDX2 in vivo*. The results showed that only *NtPDX1.3* and *NtPDX2* can form a functional complex, which is consistent with previous findings in *Arabidopsis* (Titiz et al., 2006). This suggests that in tobacco, *NtPDX1.3* and *NtPDX2* may form a functional complex to provide and accept ammonium groups, facilitating the *de novo* biosynthesis of PLP.

We cloned the *NtPDX2* from the *Nicotiana tabacum* L., constructed overexpression and gene-editing vectors for *NtPDX2*, and performed Agrobacterium-mediated transformation of tobacco leaves to generate *NtPDX2* overexpressing and knockout plants. To determine whether the expression level of the *NtPDX2* changed in the overexpression and knockout plants, we used qRT-PCR to analyze the expression levels of *NtPDX2* in stem tissues from different growth stages of the overexpressing, knockout, and control plants. We found that the expression level of *NtPDX2* was highest during the flowering stage, followed by the bolting stage, and lowest during the seedling stage. In the overexpressing plants, the mRNA expression of *NtPDX2* driven by the CaMV 35S2 promoter was significantly increased. However, there was no significant difference in the expression of *NtPDX2* between the knockout and control plants, which led to an increase in total vitamin B_6_ content in the overexpressing plants. Compared to the control, the vitamin B_6_ content in the stem tissue of the overexpressing plants was significantly higher (up to 150%), while it was reduced to 60% in the knockout plants. Earlier experiments have reported similar results. In rice, constitutive overexpression of *AtPDX1.1* and *AtPDX2* using the CaMV 35S promoter led to a substantial increase in vitamin B_6_ content in rice leaves (up to 28.3-fold), roots (up to 12-fold), and seeds (up to 3.1-fold) (Mangel et al., 2019). After overexpressing *Pdx1* and *Pdx2* isolated from *Cercospora nicotianae* in tobacco, the increase in vitamin B_6_ content was limited (Herrero and Daub, 2007). In transgenic potatoes, constitutive overexpression of *AtPDX2* driven by the CaMV 35S promoter significantly increased vitamin B_6_ accumulation in tubers (up to 107-150%) compared to non-transgenic controls (Bagri et al., 2018). Other studies have shown that overexpression of *Pdx2* alone in transgenic Arabidopsis results in a higher vitamin B_6_ content than overexpression of *Pdx1* (Chen and Xiong, 2009). These findings suggest that the biosynthetic pathway of vitamin B_6_ in plants is tightly regulated.

In our study, we measured the plant height and stem thickness of the overexpressing, knockout, and control plants at the mature stage. The results showed that the overexpressing plants were significantly taller than the control plants, while the knockout plants were noticeably shorter. The stem thickness of the overexpressing plants was significantly thicker than that of the control plants, while the stem thickness of the knockout plants was thinner. This indicates that the accumulation of vitamin B_6_ in the stem tissue of transgenic overexpressing tobacco plants leads to enhanced plant growth, resulting in taller plants and thicker stems. In contrast, the lack of PDX2 protein and vitamin B_6_ in the knockout plants inhibits their growth, leading to shorter plants and thinner stems. Previous studies have also shown that overexpression of *Pdxs* in Arabidopsis, tobacco, and potatoes results in an increase in the size of plant tissues, organs, and seeds due to the accumulation of PDX proteins and vitamin B_6_ in the plant’s sink tissues (Alonso-Blanco et al., 1999; Riefler et al., 2006; Bagri et al., 2018). Transcriptomic analysis of stem tissues from overexpressing, knockout, and control plants revealed that the expression of *NtPDX2* was significantly increased in the overexpressing plants, and vitamin B_6_ content was also significantly higher, whereas there was no noticeable difference between the knockout and control plants, but the vitamin B_6_ content in the knockout plants was much lower. We also observed that compared to the control, the expression levels of many previously reported genes that regulate plant height changed in the overexpressed (OE) and knockout (KO) plants, revealing the regulatory roles of several key genes. These genes are involved in processes such as gibberellin (GA), abscisic acid (ABA), brassinosteroid (BR) signaling pathways, cell expansion, and oxidative stress tolerance.

Gibberellin promotes plant height by relaxing the cell wall and enhancing cell elongation, particularly playing a critical role during stem elongation. In this study, we found that overexpression of the *NtPDX2* led to increased levels of vitamin B_6_ (VB6), which, in turn, resulted in the upregulation of the genes *NtGID1Cs* and *NtGAO1Ds* in the GA signaling pathway, significantly enhancing GA signal transduction. The *GID1* encodes the gibberellin receptor, which plays a crucial role in perceiving GA signals. The increased expression of *GID1* enhances the degradation of DELLA proteins, thus relieving their growth-inhibiting effect and promoting stem elongation (Griffiths et al., 2006). The *NtGAO1D* family, as a key oxidase in gibberellin biosynthesis, can enhance GA synthesis, thereby further promoting cell elongation and stem development (Appleford et al., 2006). The changes in vitamin B_6_ levels indirectly affect GA biosynthesis, thereby influencing the growth and development of tobacco stem tissue.

Auxin regulates cell elongation by acidifying the cell wall and activating cell wall loosening proteins, particularly influencing the growth of roots and stems. The SAUR (Small Auxin Upregulated RNA) gene family is an important component of the auxin signaling pathway and mediates the effects of auxin on stem elongation. For instance, in Arabidopsis, *SAUR63* is induced by auxin and promotes the elongation of hypocotyls and anther filaments. Furthermore, *SAUR63* promotes cell elongation by affecting auxin transport and response, interacting with the *PIN* and *ABCB* families of auxin efflux transporters to influence the polar transport of auxin (Chae et al., 2012). In our study, overexpression of the *NtPDX2* led to an increase in vitamin B_6_ levels in stem tissue. Transcriptomic data further revealed that the expression of *NtSAUR63* was upregulated. We hypothesize that the overexpression of *NtPDX2* indirectly affects the auxin signaling pathway through changes in vitamin B_6_ content, thereby promoting cell elongation and stem development.

Brassinosteroids (BRs) are widely distributed polyhydroxy steroid hormones in plants that have significant effects on growth, development, and stress tolerance. Studies have shown that *NtC3H15* can negatively regulate cell elongation by inhibiting the BR signaling pathway. Its downregulation removes its suppression on BES1/BZR1, leading to the upregulation of *BZR1* expression. The increased expression of *BZR1* then inhibits *IBH1* expression, resulting in a decrease in *NtIBH1* levels. *IBH1*, as a transcriptional repressor, can suppress ACEs’ DNA binding ability by interacting with them, thereby negatively regulating cell elongation. For example, overexpression of *IBH1* in Arabidopsis and rice results in dwarfism and erect leaves (Zhang et al., 2009). In this study, transcriptomic analysis indicated that the expression of *NtC3H15* decreased in *NtPDX2* overexpressing lines. We infer that the overexpression of the *NtPDX2* leads to increased vitamin B_6_ content, which subsequently inhibits the expression of *C3H15* in the BR signaling pathway, relieving its suppression of *BZR1*. The upregulation of *BZR1* further inhibits the expression of *IBH1*, thereby positively regulating cell elongation and stem development. Moreover, *NtC3H15* expression can enhance the activation of downstream genes, such as *SAUR15*, further promoting cell elongation and stem development (Chai et al., 2022). *PRE1* can interact with *IBH1* to relieve its inhibition of ACEs, restoring cell elongation (Ikeda et al., 2012). In addition, *NtBRH1* (BR-responsive RING-H2 gene) is another gene related to the BR signaling pathway, involved in plant responses to brassinosteroids. In transgenic Arabidopsis, antisense expression of *BRH1* causes thickening of the stem (Molnar et al., 2002). In summary, changes in vitamin B_6_ content may indirectly alter the BR signaling pathway, thereby regulating cell elongation and stem development, highlighting its crucial role in plant growth and development.

ABA is an endogenous plant hormone that exerts widespread effects on plant growth, development, and environmental adaptation. Moreover, ABA finely regulates plant growth and development through interactions with other hormones. The *NtP2C63*, belonging to the PP2C-D subfamily of 2C-type protein phosphatases, is a core component of the ABA signaling pathway. Three key components of ABA signal transduction have been identified in plants: ABA receptors (PYR/PYL/RCAR proteins), negative regulators (PP2C), and positive regulators (SnRK2), together forming a dual negative feedback system (PYR/PYL/RCAR-PP2C-SnRK2) to regulate ABA signal transduction and downstream responses (Soon et al., 2012). Studies have shown that PP2C-D phosphatases negatively regulate stem elongation by reducing the phosphorylation level of Thr-947 in the C-terminal self-inhibitory domain of plasma membrane H+-ATPases, thereby decreasing their activity and inhibiting cell expansion and stem elongation (Soon et al., 2012). For example, overexpression of PP2C-D1 leads to dwarfism and shorter hypocotyls (Spartz et al., 2014). In contrast, increased H+-ATPase activity promotes proton transport outside the cell, acidifying the extracellular environment, which is crucial for loosening and expanding the cell wall, ultimately facilitating stem development (Yan et al., 2024). In our study, we observed a downregulation of *NtP2C63* expression in *NtPDX2* overexpressing lines, suggesting that the increase in vitamin B_6_ levels may enhance H+-ATPase activity, thus promoting stem elongation.

The functional analysis of the *NtPDX2* in tobacco stem development revealed its complex regulatory roles in various plant hormone signaling pathways, including GA, BR, IAA, and ABA. By precisely regulating these plant hormone signaling pathways and cellular physiological processes, *PDX2* influences tobacco stem development. This finding not only elucidates the potential molecular mechanisms of *PDX2* in plant growth and development but also provides a new theoretical basis for plant growth regulation and genetic improvement. It opens new perspectives for further studies on the molecular mechanisms underlying plant growth and development and offers novel targets for genetic engineering approaches to enhance plant growth traits. Future research can further explore the functions of the *PDX2* in other plants and its regulatory mechanisms under different environmental stress conditions, providing broader prospects for plant genetic improvement and agricultural production.

## Data Availability

The original contributions presented in the study are included in the article/**Supplementary Material**. Further inquiries can be directed to the corresponding authors.
