# Supplementary material for: Functional analysis of NtPDX2 in Nicotiana tabacum L. associated with stem development
Source: Front Plant Sci. 2025 Apr 22;16:1547677. doi: 10.3389/fpls.2025.1547677 (PMC12052705; doi:10.3389/fpls.2025.1547677)
Supplement: Supplementary file 1 [file Table1.docx]

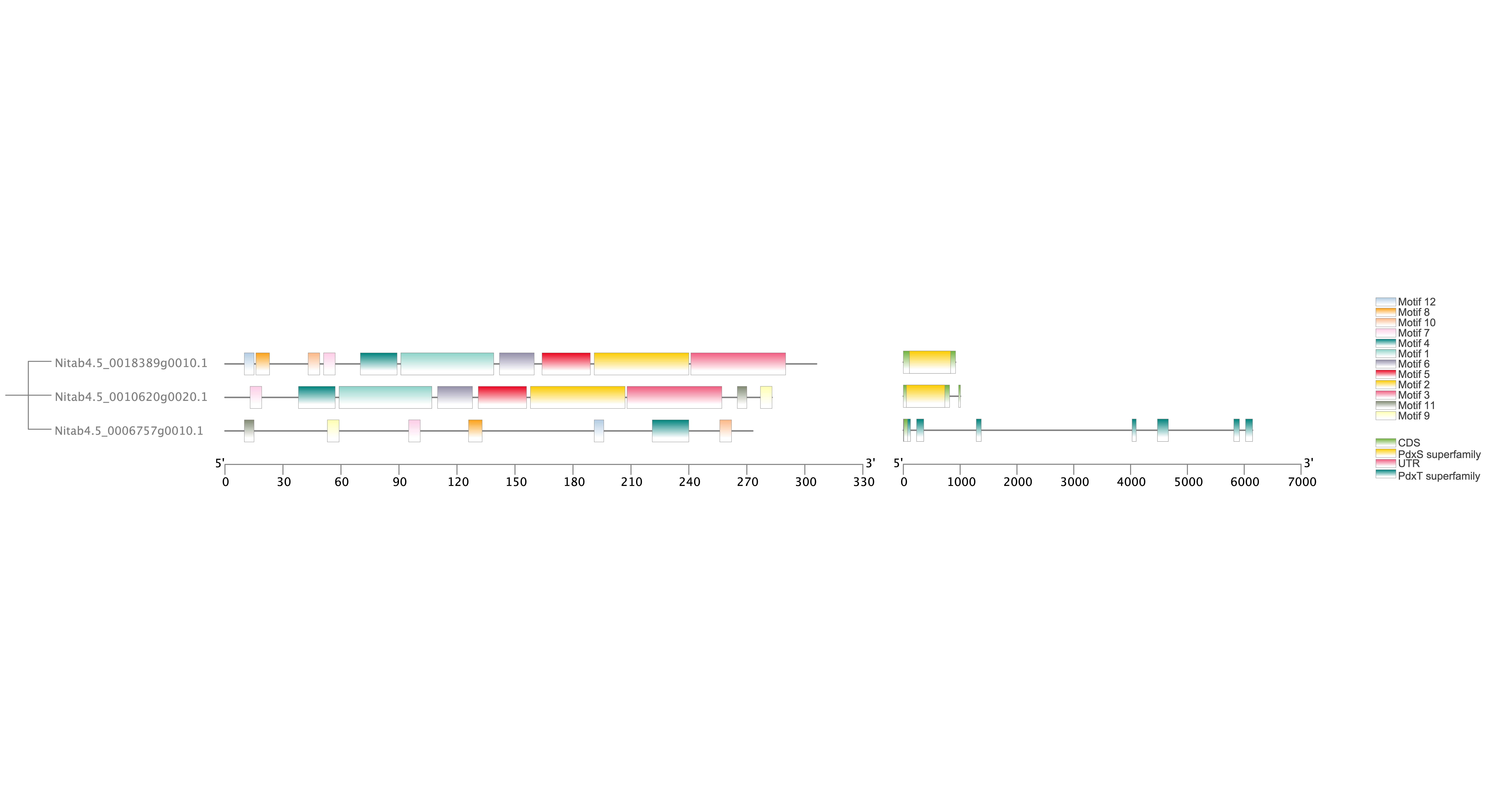


**Figure S1** Structure analysis of *Pdxs* and motif analysis of encoded protein

On the left is motif prediction analysis of PDXs protein, and on the right is structure location diagram of introns and exons of *Pdxs*.
