# Supplementary material for: Functional analysis of NtPDX2 in Nicotiana tabacum L. associated with stem development
Source: Front Plant Sci. 2025 Apr 22;16:1547677. doi: 10.3389/fpls.2025.1547677 (PMC12052705; doi:10.3389/fpls.2025.1547677)
Supplement: Supplementary file 2 [file Table2.docx]

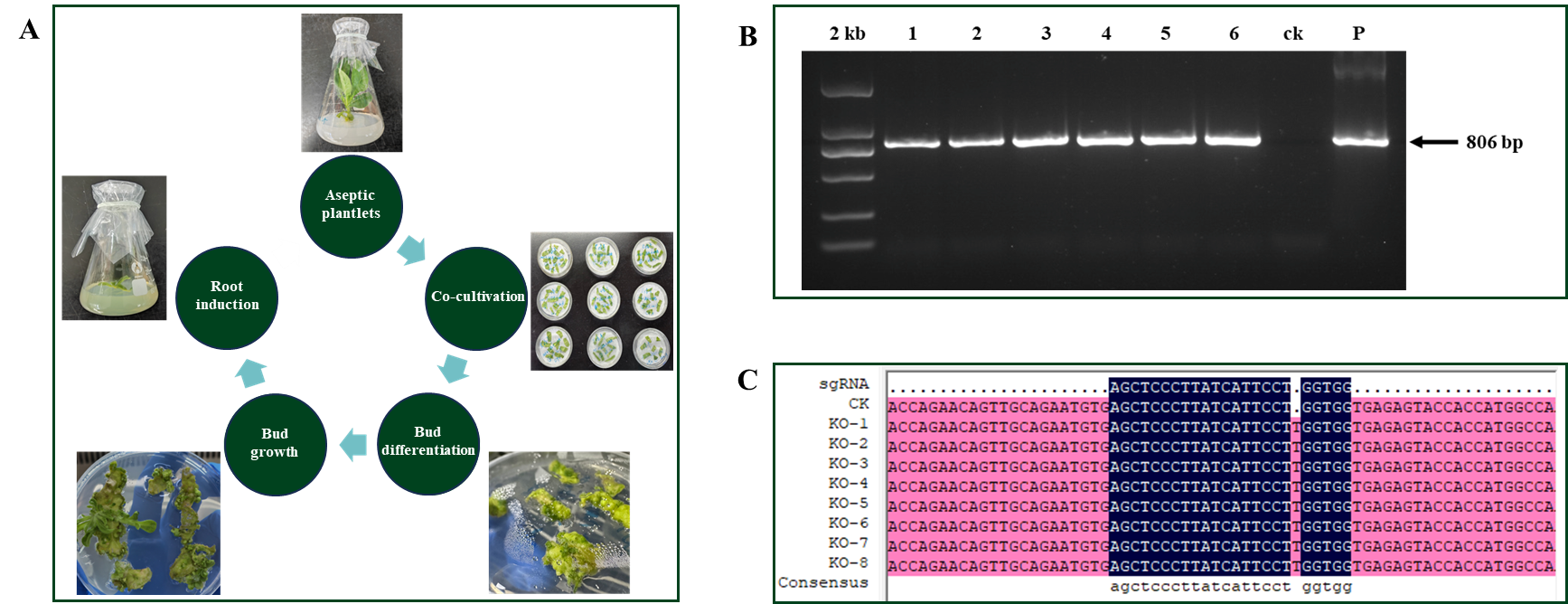


**Figure S2** Genetic transformation process of tobacco and positive plant identification of overexpression and knockout plants

A: Tobacco genetic transformation process, B: Partial PCR identification of transgenic overexpressed plants, C: Sanger sequencing identification of knockout plants.
