# Supplementary material for: Functional analysis of NtPDX2 in Nicotiana tabacum L. associated with stem development
Source: Front Plant Sci. 2025 Apr 22;16:1547677. doi: 10.3389/fpls.2025.1547677 (PMC12052705; doi:10.3389/fpls.2025.1547677)
Supplement: Supplementary file 3 [file Table3.docx]

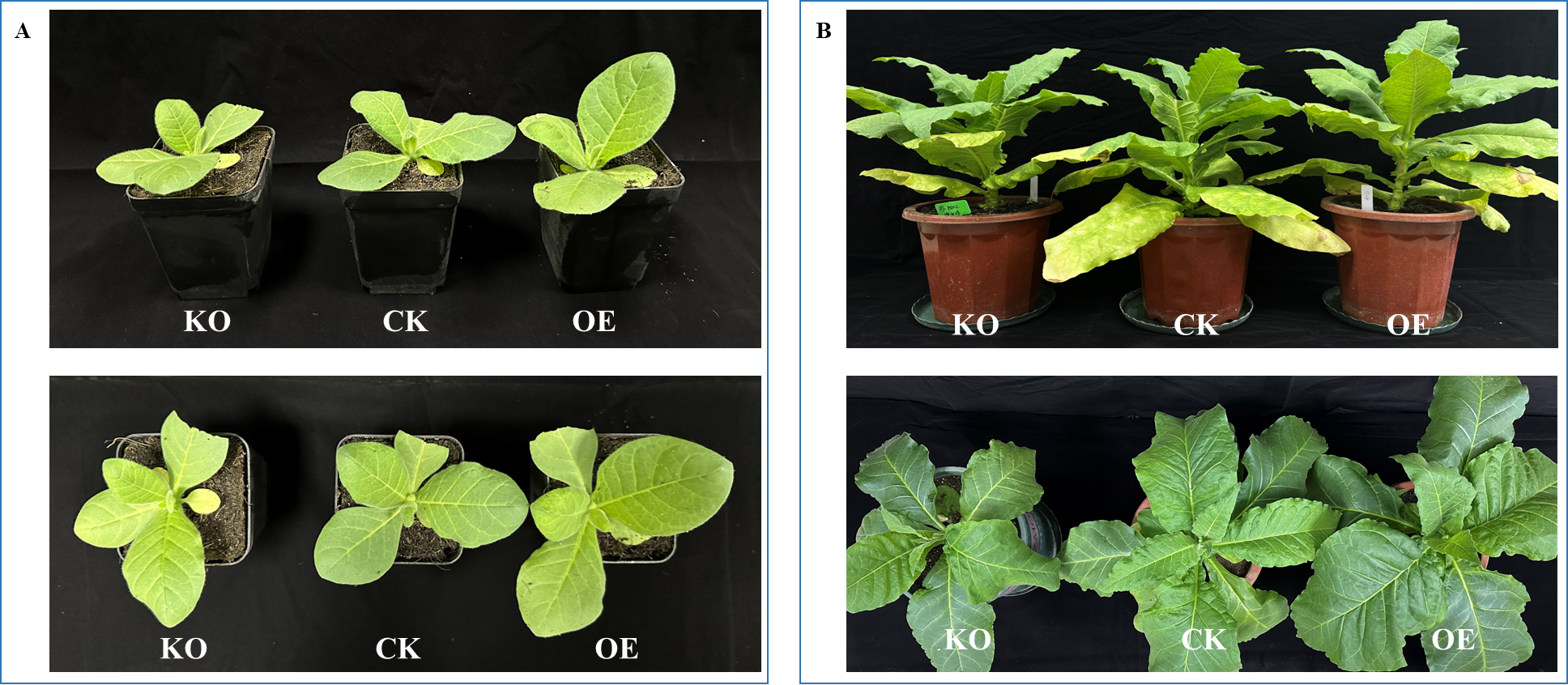


**Figure S3** Growth of different tobacco materials at seedling stage and rosette stage

A: The growth of overexpressed plants, knocked out plants and receptor plants at seedling stage; B: the growth of overexpressed plants, knocked out plants and receptor plants at rosette stage.

KO represents knockout plants, CK represents recipient plants, and OE represents overexpressed plants.
