# Supplementary material for: Functional analysis of NtPDX2 in Nicotiana tabacum L. associated with stem development
Source: Front Plant Sci. 2025 Apr 22;16:1547677. doi: 10.3389/fpls.2025.1547677 (PMC12052705; doi:10.3389/fpls.2025.1547677)
Supplement: Supplementary file 4 [file Table4.docx]

**Supplementary table 1** The sequences of primers used in this study

| **Primers** | **Sequences (5’-3’)** | **Size (bp)** | **Temperature（℃）** |
| --- | --- | --- | --- |
| **Cloning** | | | |
| NtPDX2-F1 | ATGGTTGTGGGGGTTCTTGCTT | 756 | 56 |
| NtPDX2-R1 | CTATTGGTATATGGGAAAATCAATTAT |  |  |
| NtPDX1.2-F | ATGGAAGAAGACGGTGCCGTTACAG | 921 |  |
| NtPDX1.2-R | TCAATAAGTCTCTTCTGCACTAACAAATCGCTCAACTC |  |  |
| NtPDX1.3-F | ATGGCCGGAAGCGGTGTGGTAACACT | 930 |  |
| NtPDX1.3-R | TCACTCAGAACGATTAGCATACCTCT |  |  |
| **Spilt-LUC** | | | |
| R-cLUC-2C-R | CCGATGATACGAACGAAAGCTCTGCAGTTATTGGTATATGGGAAAATCAATTATTGATCGCTCTATGAAG | 806 | 68 |
| R-cLUC-2C-F2 | TCCCGGGGCGGTACCCGGGATCATGGTTGTGGGGGTTCTTGC |  |  |
| R-1C1.2- nLUC-F | GGGACGAGCTCGGTACCCGGGATCATGGAAGAAGACGGTGCCGTTACAGTGTACAGT | 966 |  |
| R-1C1.2- nLUC-R | TCCACGCGTACGAGATCTGGTCGAATAAGTCTCTTCTGCACTAACAAATCGCTCAACTCTATTCTCGT |  |  |
| R-1C1.3-nLUC-F | GACGAGCTCGGTACCCGGGATCCATGGCCGGAAGCGGT | 972 |  |
| R-1C1.3-nLUC-R2 | CACGCGTACGAGATCTGGTCGACTCAGAACGATTAGCATACCTCTCCAC |  |  |
| **Overexpression** | | | |
| R-PDX2-F1 | CGATCGGGGAAATTCGAGCTCTTACATGGTGGTGGTGGTGGTGGCTAGATTGGTATATGGGAAAATCAATTATTGA | 825 | 68 |
| R-PDX2-R1 | TACAAATCTATCTCTGGATCCATGGTTGTGGGGGTTCTTGCT |  |  |
| **Subcellular Localization** | | | |
| R-EGFP-F | CGATCGGGGAAATTCGAGCTCTTACTTGTACAGCTCGTCCA | 762 | 68 |
| R-EGFP-R | TACAAATCTATCTCTGGATCCATGGTGAGCAAGGGCGAGGAGCT |  |  |
| R-PDX2-F | GCCCTTGCTCACCATGGATCCTTGGTATATGGGAAAATCAATTATTGA | 1521 |  |
| R-PDX2-R | TACAAATCTATCTCTGGATCCATGGTTGTGGGGGTTCTTGCT |  |  |
| **Editing Detection** | | | |
| BJJC-F | CTCTAGCGGCGTTGCGG | 620 | 58 |
| BJJC-R | CATTGGAAAGCGGTGCACGT |  |  |
| **Positive Detection（OE）** | | | |
| JC-PDX2-F2 | CTCCAATTTCAGGCACCATCTTCA | 806 | 64 |
| JC-PDX2-R2 | TCTCCACTGACGTAAGGGATGA |  |  |
| **qRT-PCR** | | | |
| q-PDX2-F1 | GCTCCACAATCTGTTCCCTGCT | 209 | 60 |
| q-PDX2-R1 | GAGGAATAGGAAGTTCTGTCTCA |  |  |
| qEF1-F | GCATTGCTTGCTTTCACCCTT | 114 |  |
| qEF1-R | AACCTCCTTCACGATTTCATCATACC |  |  |
