# Supplementary material for: Functional analysis of NtPDX2 in Nicotiana tabacum L. associated with stem development
Source: Front Plant Sci. 2025 Apr 22;16:1547677. doi: 10.3389/fpls.2025.1547677 (PMC12052705; doi:10.3389/fpls.2025.1547677)
Supplement: Supplementary file 5 [file Table5.docx]

>NtPDX2

LLTLPSFSPYPKNSSAATLFVKMVVGVLALQGSFNEHIAALKRLGVKGVEVRKPEQLQNVSSLIIPGGESTTMAKLAELHNLFPALREFVQLGKPVWGTCAGLIFLANKATGQKTGGQELIGGLDCTVHRNFFGSQIQSFETELPIPQIVAKEGGPPSFRAVFIRAPAILDVGPDVEVLADIPLSAVENINSNNAIQKEEESSESEKKVIVAVKQGNLLATAFHPELTADTRWHSYFLKMVPEIGEGTSAVISTPTTDQSFIERSIIDFPIYQ

>NtPDX1.2

MEEDGAVTVYSGSAITDTKKNPFSIKVGLAQMLRGGAIAEVTTVDQAKIAESAGACCLVVSEPIGPGISRMADPSLIKEIKQAVSIPVMAKARVGHFVEAQILEAIGADYVDESEVLALADEDHFINKHNFRAPFVCGCGDLGEALRRVREGAAMIRTQGDLLGTGNIVDTVRNVRKVMGDVRVLSNMDEDEVFTFSKKISAPYDIVAQTKQMGRLPVVHFAAGGIVTPADAALMMQLGCDGVFLGPDIFNCSDPYKKVRAIVQAVRNYNDPHILAAASSGLEEAMGGLNLNENRVERFVSAEETY

>NtPDX1.3

MEEDGAVTVYSGSAITDTKKNPFSIKVGLAQMLRGGAIAEVTTVDQAKIAESAGACCLVVSEPKGPGISRMADPSLIKEIKQAVSIPVMAKARVGHFVEAQILEAIGADYVDESEVLALADEDHFINKHNFRAPFVCGCCDLGEALRRVREGAAMIRTQGDLLGTGNIVETVRNVRKVMGDIRVLSNMDEDEVFTFSKKISAPYDIVAQTKQMGRLPVVHFAAGGIVTPADAALMMQLGCDGVFLGPDIFNCSDPYKKVRAIVQAVRNYNDPHILAAASSGLEEAMGGLNLNENRVERFVSAEETY

>GhPDX2_D05G007750

MAVVGVLALQGSFNEHIAALRRLGMKGVEIRKPEQLQSISSLIIPGGESTTMAKLAEFHNLFPALREFVQMGKPVWGTCAGLIFLANKAVGQKDGGQELVGGLNCTVHRNYFGSQIQSFEAELLVPELASQEGGPETFRGVFIRAPAVLEVGPEVEVLADYPIPSNKVLYSSSAVEIQEESAVPEKKVIVAIKQGNLLGTAFHPELTADTRWHSYFLKMVRDVGEGTSNATVAVSEAASSSDRQTKYDLPIFR

>GhPDX2_A05G007670

MAVVGVLALQGSFNEHIAALRRLGMKGVEIRKPEQLQSISSLIIPGGESTTMAKLAEFHNLFPALREFVQMGKPVWGTCAGLIFLANKAVGQKEGGQELVGGLNCTVHRNYFGSQIQSFEAELLVPELASQEGGPETFRGVFIRAPAVLEVGPEVEVLADYPIPSKKVLYSSSAVEIQEESAVPEKKVIVAIKQGNLLGTAFHPELTADTRWHSYFLKMVRDVGEGTSNTTVAVSEAASSSDRQTKYDLPIFR

>GhPDX1_A04G110900

MSDPQLIKEIKQAVTIPVMAKARIGHFVEAQILEAIGIDYVDESEVLTLADEENHINKHNFRIPFVCGCRNLGEALRRIREGAAMIRTKGEAGTGNVIEAVRHVRSVMGDIRVLRNMDDDEVFSFAKKIQSPYDLVMQTKQLGRLPVVQFAAGGVATPADAALMMQLGCDGVFVGSGVFKSGDPAKRARAIVQAVTHYSDPNMLAEVSCGLGEAMVGLNLNDKKVERFAARSDX

>GhPDX1_A10G068400

MAEDGVVTLYNNTAITDTKKNPVSIKVGLAQMLRGGAILEVSNTNQAKIAEEAGACCLAITEPNRHGISRMPDPALIKQIKRAVSIPIMSRSRVGHFVEAQILERVGVDYIDENEVLAIADEDNFINKHNFRCPFVCGCRNLGEALRRVREGAAMIRTQGDLSGTGNIVETVKNVRSVMGEIRILNNMDEDEVFAFSKKIAAPYDLVAQTKQMGRLPVVHFAAGGIVTPADAALMMQLGCDGVFVGSEVFDNCSDPYKLVHGIVEAVRHYNDPHVLVENSCGLEGEMAGLNVTEERMEQFGEX

>OsPDX2_02g03740

MAVVGVLALQGSFNEHLAALRRIGVRGVEVRKPEQLQGLDSLIIPGGESTTMAKLANYHNLFPALREFVGTGRPVWGTCAGLIFLANKAVGQKSGGQELIGGLDCTVHRNFFGSQLQSFETELSVPMLAEKEGGSDTCRGVFIRAPAILDVGSNVEVLADCPVPSDRPSITIASGEGVEEEVYSKDRVIVAVRQGNILATAFHPELTSDSRWHRFFLDMDKESDTKAFSALSLSSSSRDTQDGSKNKPLDLPIFE

>OsPDX1_07g0100200

MATDGTGVVTVYGSGTNGAALLEPSNHKSATFSVKVGLAQMLRGGVIMDVVTPEQARIAEEAGACAVMALERVPADIRAQGGVARMSDPGLIRDIKRAVTIPVMAKARIGHFVEAQILEAIGVDYVDESEVLTLADDAHHINKHNFRVPFVCGCRDLGEALRRIREGAAMIRTKGEAGTGNVVEAVRHVRSVMGDIRALRNMDDDEVFSYAKRIAAPYDLVMQTKQLGRLPVVQFAAGGVATPADAALMMQLGCDGVFVGSGIFKSGDPARRARAIVQAVTHYSDPKILAEVSSGLGEAMVGINLSDPKVERFAARSE

>AtPDX1.1_AT2G38230

MAGTGVVAVYGEGAMTETKQKSPFSVKVGLAQMLRGGVIMDVVNAEQARIAEEAGACAVMALERVPADIRAQGGVARMSDPEMIKEIKNAVTIPVMAKARIGHFVEAQILEAIGVDYVDESEVLTLADEDNHINKHNFKIPFVCGCRNLGEALRRIREGAAMIRTKGEAGTGNVVEAVRHVRSVNGAIRLLRSMDDDEVFTYAKKIAAPYDLVVQTKELGRLPVVQFAAGGVATPADAALMMQLGCDGVFVGSGVFKSGDPVKRAKAIVQAVTNYRDAAVLAEVSCGLGEAMVGLNLDDKVERFASRSE

>AtPDX1.2_AT3G16050

MADQAMTDQDQGAVTLYSGTAITDAKKNHPFSVKVGLAQVLRGGAIVEVSSVNQAKLAESAGACSVIVSDPVRSRGGVRRMPDPVLIKEVKRAVSVPVMARARVGHFVEAQILESLAVDYIDESEIISVADDDHFINKHNFRSPFICGCRDTGEALRRIREGAAMIRIQGDLTATGNIAETVKNVRSLMGEVRVLNNMDDDEVFTFAKKISAPYDLVAQTKQMGRVPVVQFASGGITTPADAALMMQLGCDGVFVGSEVFDGPDPFKKLRSIVQAVQHYNDPHVLAEMSSGLENAMESLNVRGDRIQDFGQGSV

>AtPDX1_AT5G01410

MEGTGVVAVYGNGAITEAKKSPFSVKVGLAQMLRGGVIMDVVNAEQARIAEEAGACAVMALERVPADIRAQGGVARMSDPQMIKEIKQAVTIPVMAKARIGHFVEAQILEAIGIDYIDESEVLTLADEDHHINKHNFRIPFVCGCRNLGEALRRIREGAAMIRTKGEAGTGNIIEAVRHVRSVNGDIRVLRNMDDDEVFTFAKKLAAPYDLVMQTKQLGRLPVVQFAAGGVATPADAALMMQLGCDGVFVGSGIFKSGDPARRARAIVQAVTHYSDPEMLVEVSCGLGEAMVGINLNDEKVERFANRSE

>AtPDX2_AT5G60540

MTVGVLALQGSFNEHIAALRRLGVQGVEIRKADQLLTVSSLIIPGGESTTMAKLAEYHNLFPALREFVKMGKPVWGTCAGLIFLADRAVGQKEGGQELVGGLDCTVHRNFFGSQIQSFEADILVPQLTSQEGGPETYRGVFIRAPAVLDVGPDVEVLADYPVPSNKVLYSSSTVQIQEEDALPETKVIVAVKQGNLLATAFHPELTADTRWHSYFIKMTKEIEQGASSSSSKTIVSVGETSAGPEPAKPDLPIFQ

>GmPDX2_01G116100

MAVVGVLALQGSFNEHIAALRRLGVKGVEIRKPEQLNTISSLIIPGGESTTMAKLAEYHNLFPALREFVQMGKPVWGTCAGLIFLANKAMGQKTGGQYLVGGLDCTVHRNFFGSQIQSFEAELSVPELVSKEGGPETFRGIFIRAPAILEAGPEVQVLADYLVPSSRLLSSDSSIEDKMENAEEESKVIVAVRQGNILATAFHPELTADTRWHSYFVKMSNEIGEEASSSSLVPAQVSTSQYQQPRNDLPIFQ

>GmPDX2_03G060100

MAVVGVLALQGSFNEHIAALRRLGVQGVEIRKPEQLNTISSLIIPGGESTTMAKLAEYHNLFPALREFVQMGKPVWGTCAGLIFLANKAIGQKTGGQYLVGGLDCTVHRNFFGSQIQSFEAELSVPELVSKEGGPETFCGIFIRAPAILEAGPEVQVLADYPVPSSRLLSSDSSIEDQTENAEKESKVIVAVRQGNILATAFHPELTADTRWHSYFVKMSNEIREEASSSSLVPAQVSSTSQYQQPRNDLPIYR

>GmPDX1_13G225000

MEGSGSGVVTVYGNGAITETKKSPFSVKVGLAQMLRGGVIMDVVNAEQARIAEEAGACAVMALERVPADIRAQGGVARMSDPQLIKDIKRAVTIPVMAKARIGHFVEAQILEAIGIDYVDESEVLTLADDANHINKHNFRIPFVCGCRNLGEALRRIREGAAMIRTKGEAGTGNIIEAVRHVRSVMSDIRVLRNMDDDEVFTFAKSIAAPYDLVMQTKQLGRLPVVHFAAGGVATPADAALMMQLGCDGVFVGSGVFKSGDPAKRARAIVQAVTHYSDPEVLAEVSCGLGEAMVGINLTDDKVERFANRSE

>GmPDX1_15G087200

MEGSGSGVVTVYGNGAITETKKSPFSVKVGLAQMLRGGVIMDVVDAEQARIAEEAGACAVMALERVPADIRAQGGVARMSDPQLIKDIKRAVTIPVMAKARIGHFVEAQILEAIGIDYVDESEVLTLADDANHINKHNFRIPFVCGCRNLGEALRRIREGAAMIRTKGEAGTGNIIEAVRHVRSVMSDIRVLRNMDDDEVFTFAKNIAAPYDLVMQTKQLGRLPVVHFAAGGVATPADAALMMQLGCDGVFVGSGVFKSGDPAKRARAIVQAVTHYSDPEILAEVSCGLGEAMVGINLTDDKVERFANRSE

>SlPDX2_11T002287

MVVGVLALQGSFNEHIAVLKRLGVKGVEVRKPEQLLNVSSLIIPGGESTTMAKLAELHNLFPALREFVQMGKPVWGTCAGLIFLANKATGQKTGGQKLIGGLDCTVHRNFFGSQIQSFETELPIPQVVAEDGGPPSFRAVFIRAPAILDVGPDVEVLSDIPLSAIETLNSNPAIQKEEESTESGKKVIVAVKQGNLLATAFHPELTADTRWYVSSATSMNVIYYLSGTMHSYFLKMVPEIEGGTSDIVSTSTSNQSFGTRSIIDFPIYQ

>SlPDX1_03T003305

MEEDGAVTVYSGSAITDTKKNSFSIKVGIAQMLRGGAIAEVTTVNQAKIAESAGVCCLVVSEPKGPGISRMPDPSLIKEIKQAVALPVMAKARVGHFVEAQILEAIGVDYIDESEILALADEDHFVNKHNFRAPFVCGCRDLGEALRRVREGAAMVRTQGDLAGTGSIVDTVHNVRKVMGDIRILSNMDDDEVFTFSKKIGAPYDIVAQTKQMGRLPVVHFAAGGIVTPADAALMMQLGCDGVFVGSDIFNCSDPYKKVRAIVQAVRNYNDPHILAAASSGLEEAMGGLNLNENRVERFVSDENY

>StPDX2_11G022660

MVVGVLALQGSFNEHIAVLKRLGVKGVEVRKPEQLLNVSSLIIPGGESTTMAKLAELHNLFPALREFVQMGKPVWGTCAGLIFLANRATGQKTGGQKLIGGLDCTVHRNFFGSQIQSFETELPIPQVVAEEGGPPSFRAVFIRAPAILDVGPDVEVLADIPLSAIETINSNPAIPKEEDSTESGKKVIVAVKQGNLLATAFHPELTADTRWHSYFLKMVPEIEEGTSDIVSTSTSNQSFGARSIIDFPIYQ

>StPDX1_06G032300

MAGSGVVTVYGNGALTETTKQSPFSVKVGLAQMLRGGVIMDVVNAEQARIAEEAGACAVMALERVPADIRAQGGVARMSDPQLIKEIKQAVTIPVMAKARIGHFVEAQILEAIGVDYVDESEVLTLADDENHINKHNFRIPFVCGCRNLGEALRRIREGAAMIRTKGEAGTGNIIEAVRHVRSVMGDIRVLRNMDDDEVFTFAKKIQAPYDLVMQTKQLGRLPVVHFAAGGVATPADAALMMQLGCDGVFVGSGIFKSGDPAKRGRAIVQAVTHYSDPQLLAEISCGLGEAMVGINLDEKVERYANRSE

>MePDX2_04G022200

MAVGVLALQGSFNEHIAVLRRLGVKGVEIRKPEQLQDVSYLIIPGGESTTMAKLAEYHNLFPALREFVKTGKPVWGTCAGLIFLADKAIGQKTGGQELVGGLDCTVHRNYFGSQIQSFEADLLVPELVSKEGGPETFRGVFIRAPAVLEVGPGVDVLAEYPVSSTNVLYSSSAVQIQEENAVPEKKVVVAVKQGNLLGTAFHPELTADTRWHSYFLKMGSEAQEEASSSIVPVGVDLSFNEKARIDLPIYQ

>MePDX1_09G105200

MAGTGVVAVYGNGAITETKKSPFSVKVGLAQMLRGGVIMDVVNPEQARIAEEAGACAVMALERVPADIRAQGGVARMSDPQLIKEIKQSVTIPVMAKARIGHFVEAQILEAIGIDYVDESEVLTLADEENHINKHNFRIPFVCGCRNLGEALRRIREGAAMIRTKGEAGTGNVIEAVRHVRSVMGDIRVLRNMDDDEVFTFAKKIAAPYDLVMQTKQLGRLPVVQFAAGGVATPADAALMMQLGCDGVFVGSGVFKSGDPARRARAIVQAVTHYSDPDMLAEVSCGLGEAMVGINLNDKKVERFANRSE
