# Supplementary material for: Functional analysis of NtPDX2 in Nicotiana tabacum L. associated with stem development
Source: Front Plant Sci. 2025 Apr 22;16:1547677. doi: 10.3389/fpls.2025.1547677 (PMC12052705; doi:10.3389/fpls.2025.1547677)
Supplement: Supplementary file 6 [file Table6.docx]

**Supplementary table 3**

Distribution of transcript expression (FPKM) in stem tissues from different tobacco materials

| **Sample** | **FPKM>0** | **FPKM>1** | **FPKM>10** | **FPKM>100** | **All genes** |
| --- | --- | --- | --- | --- | --- |
| KO-S1 | 40739(48.35%) | 29068(34.50%) | 12370(14.68%) | 2082(2.47%) | 84259 |
| KO-S2 | 40085(48.05%) | 28839(34.57%) | 12452(14.93%) | 2051(2.46%) | 83427 |
| KO-S3 | 40863(48.45%) | 28928(34.33%) | 12389(14.70%) | 2094(2.48%) | 84274 |
| CK-S1 | 40650(45.61%) | 32096(36.01%) | 14272(16.01%) | 2104(2.36%) | 89122 |
| CK-S2 | 39541(45.91%) | 31490(36.56%) | 13097(15.21%) | 2000(2.32%) | 86128 |
| CK-S3 | 37921(46.61%) | 28970(35.61%) | 12440(15.29%) | 2019(2.48%) | 81350 |
| OE-S1 | 40333(48.36%) | 28546(34.23%) | 12544(15.04%) | 1982(2.38%) | 83405 |
| OE-S2 | 40496(50.18%) | 26997(33.45%) | 11177(13.85%) | 2039(2.53%) | 80709 |
| OE-S3 | 37615(48.51%) | 26315(33.94%) | 11454(14.77%) | 2161(2.79%) | 77545 |
